# Persistent neurological and behavioral alterations after SARS-CoV-2 infection in an optimized K18-hACE2 mouse model

**DOI:** 10.3389/fmicb.2026.1871084

**Published:** 2026-06-26

**Authors:** Carla Ruiz-Casas, Ferran Tarrés-Freixas, Núria Roca, Mònica Pérez, Estefania Contreras, Laura Martín, Olga Bernaus, Alex Olvera, Marta Ruiz-Riol, Christian Brander, Carla Usai, Júlia Vergara-Alert, Joaquim Segalés

**Affiliations:** 1 Unitat Mixta d’Investigació IRTA-UAB en Sanitat Animal, Centre de Recerca en Sanitat Animal (CReSA), Campus de la Universitat Autònoma de Barcelona (UAB), Bellaterra, Spain; 2 IRTA Programa de Sanitat Animal, Centre de Recerca en Sanitat Animal (CReSA) - Campus de la UAB, Bellaterra, Spain; 3 Biosciences Department, Faculty of Sciences and Technology, University of Vic-Central University of Catalonia, Vic, Barcelona, Spain; 4 Irsi-Caixa, Hospital Germans Trias i Pujol, Badalona, Spain; 5 CIBERINFEC, Instituto de Salud Carlos III, Madrid, Spain; 6 ICREA, Barcelona, Spain; 7 Medical Faculty, University of Vic-Central University of Catalonia, Vic, Barcelona, Spain; 8 Department de Sanitat i Anatomia Animals, Facultat de Veterinària - Campus de la UAB, Bellaterra, Spain

**Keywords:** behavioral impairment, immune dysregulation, K18-hACE2 mouse model, long COVID, neurological sequelae, post- COVID-19 condition (PCC), SARS-CoV-2, sex differences

## Abstract

**Introduction:**

Persistent neurological symptoms are among the most prevalent and debilitating manifestations of post-COVID-19 Condition (PCC), affecting millions of individuals worldwide. PCC is a chronic multisystemic syndrome that develops in over 30% of adults following acute infection with severe acute respiratory syndrome coronavirus 2 (SARS-CoV-2). Despite its major clinical and socioeconomic impact, the biological mechanisms underlying PCC remain poorly understood, underscoring the need for robust and translational animal models.

**Methods:**

We conducted a longitudinal study in K18-hACE2 mice, integrating virological, immunological, histopathological, and behavioral assessments from acute infection through 60 days post-inoculation.

**Results:**

SARS-CoV-2-inoculated mice developed persistent neurobehavioral impairments despite the absence of detectable viral replication in the brain. These alterations were associated with sustained immune dysregulation in both pulmonary and neural tissues, persistent pulmonary pathology, and a reduction in vagus nerve cross-sectional area. Notably, several long-term outcomes exhibited marked sex-dependent differences that mirrored clinical observations in human PCC.

**Discussion:**

Our findings demonstrate that K18-hACE2 mice recapitulate key neurological and immunopathological features of PCC and may constitute a valuable experimental model for investigating the mechanisms underlying PCC-associated neurological sequelae. This model may also facilitate the development and preclinical evaluation of targeted therapeutic interventions.

## 1 Introduction

As of June 2026, severe acute respiratory syndrome coronavirus 2 (SARS-CoV-2) has caused more than 779 million confirmed cases of Coronavirus Disease 2019 (COVID-19) worldwide (WHO, 2026). Although most individuals recover from the acute phase of infection, a substantial proportion subsequently develop persistent or recurrent symptoms collectively referred to as post-COVID-19 condition (PCC) or Long COVID, affecting an estimated 30%–40% of infected adults (Hou et al., 2025).

PCC is a chronic, multisystemic, and heterogeneous disorder characterized by symptoms that persist for at least 3 months and may follow continuous, relapsing–remitting, or progressive clinical courses. Clinical manifestations can involve multiple organ systems, including the respiratory, cardiovascular, gastrointestinal, musculoskeletal, and nervous systems (Ely et al., 2024). Among these, neurological and neuropsychiatric symptoms (neuro-PCC) are particularly common and disabling, frequently including cognitive impairment, fatigue, anxiety, depression, sleep disturbances, autonomic dysfunction, and reduced quality of life (Charles James et al., 2025; Elboraay et al., 2025; Moen et al., 2025). Despite increasing clinical recognition, the mechanisms driving neuro-PCC remain poorly understood, and effective targeted therapies are still lacking (Moen et al., 2025). Current evidence suggests that neuro-PCC may result from chronic, low-grade neuroinflammation sustained by multiple interacting processes, including viral neuroinvasion, persistence of viral RNA or antigens, prolonged systemic inflammation and tissue injury, and vagus nerve dysfunction, affecting both the peripheral and central nervous systems (Peluso and Deeks, 2024; Komaroff and Dantzer, 2025; Moen et al., 2025; Ramli et al., 2025).

Animal models are therefore essential to investigate the biological mechanisms underlying neuro-PCC and to support the development of diagnostic and therapeutic strategies. In particular, models that recapitulate acute respiratory infection followed by long-term neurological and systemic alterations without viral replication in the brain are especially valuable (Stein et al., 2022; Usai et al., 2023; Vanderheiden and Diamond, 2025). Mouse models provide important advantages because of the availability of extensive genetic, immunological, and neurobiological tools (Mukherjee et al., 2022). However, ancestral SARS-CoV-2 strains do not efficiently bind murine angiotensin-converting enzyme 2 (ACE2), requiring approaches such as human ACE2 (hACE2) transgenic expression, adeno-associated virus-mediated hACE2 delivery (AAV-hACE2), mouse-adapted (MA) viral strains, or variants carrying spike (S) mutations that enhance receptor binding (e.g., Alpha, Beta, Delta, Gamma, Omicron) (Chu et al., 2022; Tarrés-Freixas et al., 2022; Vanderheiden and Diamond, 2025). Among available models, transgenic mice expressing hACE2 under the human keratin 18 promoter (K18-hACE2) have been widely used because they support robust infection with ancestral SARS-CoV-2 strains. However, these mice typically develop severe, dose-dependent disease with high mortality driven by neuroinvasion, limiting their utility for long-term studies (Vidal et al., 2022). Consequently, optimization of the inoculation dose is necessary to balance survivability with clinically relevant disease severity and thereby enable investigation of post-acute sequelae.

Several previous studies have explored long-term outcomes after SARS-CoV-2 infection in mice using distinct experimental systems. K18-hACE2 mice inoculated with the Delta variant developed persistent immune activation and cognitive impairment up to 1 month post-inoculation (Singh et al., 2024). In AAV-hACE2 mice challenged with the ancestral B.1 strain, long-lasting neuroinflammatory changes, white-matter loss, reduced neurogenesis, and elevated cerebrospinal fluid cytokine levels (CXCL10, CCL7, CCL2, CCL11, GMCSF, IL-10, and CCL5) were observed up to 7 weeks post-inoculation (Fernández-Castañeda et al., 2022). Similarly, wild-type mice inoculated with MA10 or B.1.351 strains exhibited persistent astrogliosis in the hippocampus and/or brainstem lasting at least 28 days, with B.1.351 additionally inducing hippocampal IL-6 upregulation, impaired neurogenesis, neuronal dysfunction, and memory deficits (Fernández-Castañeda et al., 2022; Amruta et al., 2023; Singh et al., 2024; Vanderheiden et al., 2024). Collectively, these studies demonstrate that SARS-CoV-2 infection can induce durable neurological alterations across multiple mouse models. However, differences in viral strain, host susceptibility, and disease severity complicate direct comparisons and highlight the need for models optimized specifically for longitudinal PCC studies.

In the present study, we investigated post-acute sequelae in K18-hACE2 mice inoculated with a very low dose of SARS-CoV-2 B.1 strain. This approach was designed to reduce acute mortality while preserving clinically relevant acute disease, thereby enabling extended longitudinal analysis. Because early SARS-CoV-2 variants and severe COVID-19 are recognized risk factors for PCC in humans, this model may more closely reflect conditions associated with persistent neurological outcomes (Lok et al., 2024; Moen et al., 2025). We conducted the longest follow-up to date of central and peripheral neurological outcomes in this model, integrating virological, immunological, histopathological, and behavioral assessments at the individual level to characterize the heterogeneity of post-acute outcomes.

Overall, the aim of this study was to determine whether mild-to-moderate acute SARS-CoV-2 infection in K18-hACE2 mice results in sustained neurological and systemic alterations consistent with PCC. Using this optimized model, we demonstrated persistent behavioral impairments in the absence of detectable viral replication in the brain, accompanied by prolonged pulmonary and neural immune dysregulation and reduced vagus nerve cross-sectional area. The animal model presented here represents a relevant experimental framework to investigate PCC pathogenesis and support the development of targeted therapeutic strategies.

## 2 Methods

### 2.1 Ethics statement

All experimental protocols were approved by the Institutional Animal Welfare Committee of the *Institut de Recerca i Tecnologia Agroalimentàries* (CEEA-IRTA, registration number CEEA 384-2023) and by the Ethical Commission of Animal Experimentation of the Government of Catalonia (ethical approval number CEA-OH/12097/2).

### 2.2 Animal model

All experiments were conducted using 5–6-week-old B6.Cg-Tg(K18-ACE2)2Prlmn/J (K18-hACE2) mice (Charles River Laboratories). Both the challenge dose optimization (Study 1) and the longitudinal follow-up study (Study 2) were performed in biosafety level 3 (BSL-3) facilities at the IRTA-CReSA Biocontainment Unit (Barcelona, Spain), and all procedures were conducted by certified personnel in accordance with institutional and national biosafety regulations.

### 2.3 Virus isolates and cell cultures

The SARS-CoV-2 isolate Cat02 (GISAID ID: EPI_ISL-471472), belonging to the B.1 lineage, was used. The isolate was obtained from a nasopharyngeal aspirate collected from a laboratory-confirmed COVID-19 patient in Barcelona, Spain, in 2020. Relative to the Wuhan reference strain, this isolate contained the following mutations: D614G (Spike), K837N (NSP3), and P323L (NSP12).

Virus stocks (passage 3) were propagated and titrated in Vero E6 cells (ATCC® repository, CRL-1586™). Viral titers were determined using the Reed–Muench method and expressed as tissue culture infectious dose 50 (TCID_50_)/mL (Reed and Muench, 1938). Virus stock preparation and cell culture were performed as previously described (Brustolin et al., 2021). Briefly, Vero E6 cells were maintained in Dulbecco’s Modified Eagle Medium (DMEM) supplemented with 5% fetal bovine serum (FBS), 100 U/mL penicillin, 100 mg/mL streptomycin, and 2 mM glutamine.

### 2.4 Study design

A total of 120 K18-hACE2 mice were used across two studies. Animals were acclimatized for 1 week prior to experimentation, randomly assigned to experimental groups, and housed in ventilated cages (3–6 mice/cage, or individually during behavioral testing) under controlled conditions (21 ± 1 °C, 50%–60% relative humidity, 12 h light/dark cycle) and *ad libitum* access to food and water.

#### 2.4.1 Study 1: challenge dose optimization

A dose-escalation study was conducted to identify a SARS-CoV-2 inoculation dose that ensured consistent infection while minimizing mortality, thereby enabling long-term follow-up. Accordingly, 16 mice were randomly assigned to four groups (*n* = 4/group; equal sex ratio) and intranasally inoculated under isoflurane anesthesia with 10^3^, 10^2^, 10^1^, or 5 × 10^0^ TCID_50_ SARS-CoV-2 in a total volume of 50 μL (25 μL per nostril).

Animals were monitored daily for survival, body weight, and viral shedding in oropharyngeal swabs up to 11 days post-inoculation (dpi). Mice reaching humane endpoint (HEP) criteria were euthanized by intraperitoneal pentobarbital injection.

#### 2.4.2 Study 2: Long-term follow-up

Based on Study 1 results, 10^1^ TCID_50_ was selected for the longitudinal study (Study 2), as it reliably induced measurable clinical disease while limiting mortality. Preservation of measurable acute clinical signs was considered essential because greater acute COVID-19 severity is associated with an increased risk of PCC in humans (Escrivá et al., 2025).

Study 2 included 104 mice: 26 negative controls (G1; 13 females and 13 males) inoculated with PBS and 78 mice (G2; 40 females and 38 males) inoculated with 10^1^ TCID_50_ SARS-CoV-2, using a total volume of 50 μL (25 μL per nostril).

**Figure 1:**
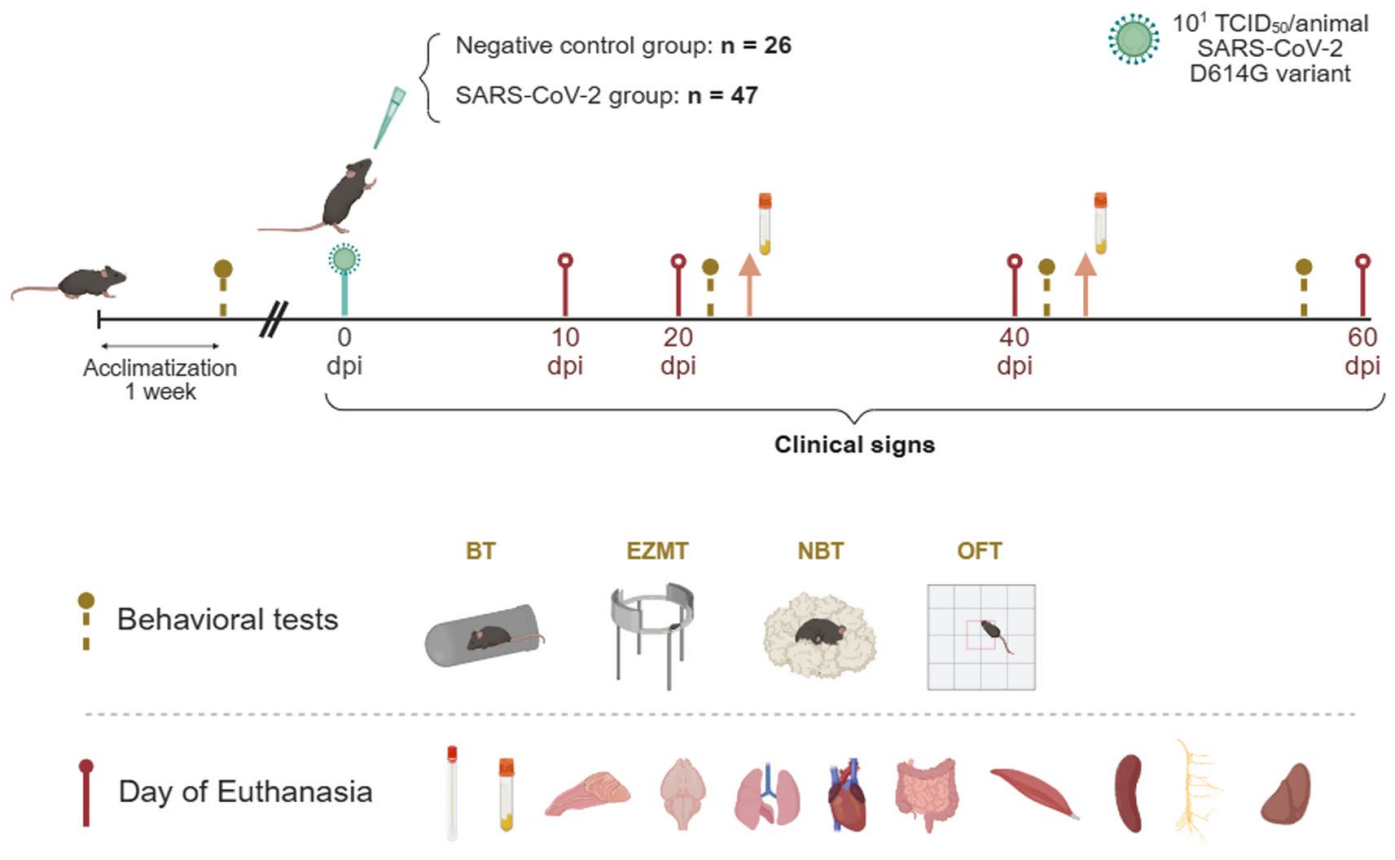
Study design and timeline for long-term monitoring after SARS-CoV-2 inoculation in K18-hACE2 mice. Forty-seven SARS-CoV-2-inoculated and 26 negative control K18-hACE2 mice were necropsied at 10, 20, 40, or 60 days post-inoculation (dpi). Oropharyngeal swabs, plasma, nasal turbinate, lung, brain, heart, intestine, muscle, spleen, vagus nerve, and liver were collected for virological, immunological and/or histopathological outcomes. Clinical monitoring included neurological signs, locomotion, breathing, behavior, appearance, body weight, and subcutaneous temperature. Mice euthanized at 60 dpi (negative control: *n* = 8; SARS-CoV-2: *n* = 25) additionally underwent longitudinal behavioral testing before inoculation and at 21, 42, and 56 dpi using the burrowing test (BT), elevated zero maze test (EZMT), nest building test (NBT), and open field test (OFT). Created with https://BioRender.com.

The experimental design timeline for Study 2 is illustrated in Figure 1, which includes only the animals that survived the acute infection and were therefore included in all longitudinal analyses (G1: *n* = 26; G2: *n* = 47). Animals reaching humane endpoint (HEP) criteria during the acute phase were excluded from the study and are not part of the experimental cohort analyzed herein. Clinical signs, body weight, and subcutaneous temperature were monitored before inoculation, daily through 10 dpi, twice weekly from 11 to 21 dpi, and weekly thereafter. Subcutaneous temperature was recorded using microchips (UCT-2112-24, Unified Information Devices) implanted under isoflurane anesthesia, and a handheld reader (URH-1HP Reader, Unified Information Devices).

Surviving mice were randomly assigned to necropsy at 10 dpi (G1: *n* = 6, 3 females and 3 males; G2: *n* = 6, 3 females and 3 males), 20 dpi (G1: *n* = 6, 3 females and 3 males; G2: *n* = 8, 5 females and 3 males), 40 dpi (G1: *n* = 6, 3 females and 3 males; G2: *n* = 8, 4 females and 4 males), or 60 dpi (G1: *n* = 8, 4 females and 4 males; G2: *n* = 25, 15 females and 10 males). The 10-dpi time point was used to evaluate acute disease in recovering mice, whereas time points ≥ 21 dpi were used to assess post-acute and PCC-like outcomes.

Mice assigned to the 60-dpi endpoint underwent longitudinal behavioral testing at baseline (pre-inoculation), 21, 42, and 56 dpi using the nest building test (NBT), burrowing test (BT), open field test (OFT), and elevated zero maze test (EZMT). Blood samples (150 μL) were collected from the saphenous vein under isoflurane anesthesia following behavioral testing at 21 and 42 dpi. All experimental procedures, data acquisition, and downstream analyses were performed under randomized conditions.

### 2.5 Clinical scoring and HEP criteria

Clinical disease severity was evaluated through standardized observational assessments using a semi-quantitative scoring system. Briefly, six clinical domains were systematically assessed: neurological signs, locomotion, breathing, behavior, appearance, and body weight. Each parameter was independently scored on a scale ranging from 0 (normal) to 3 (severe), with higher scores reflecting increasing severity of the corresponding clinical feature. The specific observational criteria used to assign each score for each parameter are provided in Supplementary Table S1. The total clinical score for each animal was calculated as the sum of all individual parameter scores, generating a composite index of disease severity. Animals were humanely euthanized upon reaching HEP criteria, defined as either a score of 3 in any individual parameter or a total clinical score ≥ 10 (Supplementary Table S1). Euthanasia was performed by intraperitoneal pentobarbital injection or by cervical dislocation, in accordance with institutional ethical guidelines.

### 2.6 Behavioral testing protocols

#### 2.6.1 General procedures

Neurocognitive performance was assessed longitudinally in 33 mice (G1: *n* = 8, G2: *n* = 25), all of which were euthanized at the final study endpoint (60 dpi). Behavioral assessments were conducted at 21, 42, and 56 dpi using a battery of four tests: BT, EZMT, NBT, and OFT.

To habituate mice to the testing environment, animals were individually housed for 72 h before testing in cages containing minimal bedding (~2 cm depth). Mice were additionally habituated to the burrowing apparatus and nestlet materials for 48 h before behavioral assessment to promote spontaneous behavior (Deacon, 2012). Behavioral testing was performed over two consecutive days at each time point. On day 1, mice underwent the BT and EZMT, after which a pressed cotton square (nestlet; NES3600, Ancare) was provided for the NBT. On day 2, nest quality was scored, followed by the OFT. Upon completion of the testing battery, animals were returned to group housing. All assessments were conducted under dim lighting conditions (70% reduction) by the same observers throughout the study. To minimize potential cross-contamination effects, negative control animals were always tested prior to SARS-CoV-2-inoculated animals and the equipment was cleaned with 5% ethanol between animals and disinfected with PeraSafe™ between testing sessions.

#### 2.6.2 Nest-building test

The BT was used to evaluate motor function and species-specific digging activity, an innate behavior which may be altered in neurodegenerative disorders (Deacon and Rawlins, 2005; Deacon, 2006b, 2012). Mice were provided with a PVC tube (4 cm diameter, 15 cm length), sealed at one end and elevated 3 cm above the cage floor, containing 45 g of clay balls. Burrowing performance was quantified as the percentage of material displaced after 30 min (Deacon, 2006b) (Supplementary Figure S1).

#### 2.6.3 Elevated zero maze test

The EZMT was used to assess exploratory and anxiety-like behaviors (Shepherd et al., 1994). Mice were placed on an elevated circular maze (100 cm diameter, 50 cm above floor level) consisting of two enclosed (anxiolytic) and two open (anxiogenic) quadrants and recorded for 7 min (Supplementary Figure S1). Behavior was analyzed using Ethovision XT 17.0 (Noldus). Outcome measures included the frequency of entries into the open zones (exploration and risk-assessment) and time spent in the open arms (anxiety-like behavior).

#### 2.6.4 Nest-building test

The NBT was used to assess innate behavior and general well-being (Deacon, 2006a, 2012). Mice were provided with a nestlet for 24 h (Supplementary Figure S1), after which nests were scored using a 5-point scale: 1 = untouched; 2 = 50%–90% intact; 3 = 50%–90% shredded without a distinct nest site; 4 = > 90% shredded forming a flat nest; 5 = > 90% shredded forming a crater-shaped nest (Deacon, 2006a).

#### 2.6.5 Open field test

The OFT was used to assess locomotor, exploratory, and anxiety-like behaviors (Seibenhener and Wooten, 2015). Mice were individually placed in the center of a white opaque square methacrylate arena (50 cm diameter x 40 cm height) and recorded for 10 min (Supplementary Figure S1). Behavior was analyzed using Ethovision XT 17.0 (Noldus). Outcome measures included total distance moved (locomotor activity), center-to-total distance ratio (exploratory and anxiety-like behavior), and time spent in the center zone (anxiety-like behavior).

#### 2.6.6 Behavioral data processing

Data analyses were performed by investigators blinded to both experimental group allocation and time point to minimize potential bias during data acquisition and interpretation.

Behavioral changes at the group level were calculated relative to baseline and expressed as delta (Δ) scores, defined as:


Δ=post−challenge value−pre−challenge value

Negative Δ values indicated reduced performance relative to baseline.

To evaluate behavioral changes at the individual level, z-scores were calculated. Prior to normalization, the distribution of behavioral data was assessed to ensure compatibility with parametric standardization. The z-score is a dimensionless normalization metric widely used in animal behavioral studies (Guilloux et al., 2011). For each behavioral parameter, the z-score for an individual animal was calculated as:


z−score=(X−μ)/σ

Where *X* represents the individual Δ value for the parameter of interest, and *μ* and *σ* correspond to the mean and standard deviation of the control group, respectively. Thus, the z-score reflects the number of standard deviations (*σ*) by which an individual observation (*X*) deviates from the mean of the control group (*μ*). Negative z-score values indicated poorer performance relative to controls, and *z*-scores < −1.96 were considered indicative of impairment, corresponding to the conventional two-tailed 95% confidence threshold under the assumption of normality (Guilloux et al., 2011).

Together, the use of Δ scores and *z*-score normalization minimized baseline-related effects and accounted for inter-individual variability, thereby improving comparability across subjects.

### 2.7 Sample collection, processing, and storage

For virological and immunological analyses, plasma, oropharyngeal swabs, and tissue samples were collected, including the left nasal turbinate, left caudal lung lobe, left caudal hemibrain, heart, small intestine, right hindlimb muscle, liver, and spleen.

Blood samples were collected either from the saphenous vein using 200 μL capillary tubes (Microvette® 200 EDTA K3E) or by cardiac puncture at necropsy. Plasma was separated by centrifugation (1000×*g*, 10 min), transferred into 1.5 mL cryotubes, and stored at −80 °C until analysis.

Oropharyngeal swabs and all tissue samples, except spleens, were collected in 2 mL cryotubes containing 1 mL DMEM supplemented with 100 U/mL penicillin, 100 μg/mL streptomycin, and 2 mM glutamine. Swabs were vortexed for 30 s before processing. Tissue samples were homogenized using a 3 mm tungsten bead (69,997, QIAGEN) in a TissueLyser II (QIAGEN) at 25 Hz for 30 s, followed by centrifugation at 2,000×*g* for 2 min. Supernatants were stored at −80 °C until further analysis.

Spleens were collected in RPMI 1640 medium supplemented with 20% FBS, 1% glutamine, and 1% penicillin/streptomycin. Splenocytes were isolated by mechanical dissociation through a 40 μm strainer, followed by red blood cell lysis using ammonium chloride buffer (17 mM TRIS, 0.14 M NH_4_Cl, pH 7.4). After washing, splenocytes were resuspended in FBS containing 10% dimethyl sulfoxide (DMSO), aliquoted into cryovials, gradually frozen at −80 °C overnight, and subsequently transferred to liquid nitrogen (−196 °C) for long-term storage.

For histopathological analyses, the right nasal turbinate, right lung, right hemibrain, heart, small intestine, right hindlimb muscle, cervical region containing the vagus nerve, and liver were collected and immersion-fixed in 10% neutral buffered formalin for 7 days before processing.

### 2.8 RNA-extraction and SARS-CoV-2 detection by RT-qPCR

Viral RNA was extracted from oropharyngeal swabs and tissue samples (nasal turbinate, lung, brain, heart, intestine, muscle, and liver) using the IndiMag Pathogen Kit (Indical Bioscience) on a Biosprint 96 workstation (QIAGEN), according to the manufacturer’s instructions.

SARS-CoV-2 genomic RNA (gRNA) was detected by real-time quantitative PCR (RT-qPCR), as previously described (Corman et al., 2020), targeting the upstream envelope (UpE) gene (GenBank NC_004718, positions 26,141–26,253). RT-qPCR was conducted using AgPath-ID™ One-Step RT-PCR Reagents (Applied Biosystems) with the following oligonucleotides: forward primer 5′-ACAGGTACGTTAATAGTTAATAGCGT-3′ (400 nM), reverse primer 5′-ATATTGCAGCAGTACGCACACA-3′ (400 nM), and probe 5′-FAM-ACACTAGCCATCCTTA CTGCGCTTCG-TAMRA-3′ (200 nM).

Thermal cycling conditions consisted of reverse transcription at 50 °C for 10 min, initial denaturation at 95 °C for 10 min, followed by 45 cycles of 94 °C for 15 s and 58 °C for 30 s, using a 7,500 Fast Real-Time PCR System (Applied Biosystems).

Absolute quantification was performed using a standard curve generated from serial 10-fold dilutions of an E gene plasmid control (10006896, 2019-nCoV_E_Positive Control, IDT), run in parallel with each assay. All samples were analyzed in duplicate, and cycle threshold (Ct) differences of < 1 cycle between replicates were considered acceptable.

### 2.9 Histological and immunological analyses

Fixed tissues, including the nasal turbinate, brain, lung, heart, intestine, muscle, liver, and cervical region, were processed into formalin-fixed, paraffin-embedded sections and cut at 4 μm thickness. Nasal turbinate and cervical region samples were decalcified prior to processing.

Hematoxylin and eosin (H&E) staining was performed on all sections to assess SARS-CoV-2-associated histopathological lesions. Lesions were scored semi-quantitatively on a 0–3 scale, as previously described (Brustolin et al., 2021; Vidal et al., 2022): 0 = absent, 1 = mild, 2 = moderate, and 3 = severe. Immunohistochemistry (IHC) for SARS-CoV-2 nucleocapsid (N) protein was performed on sections from nasal turbinate, lung, brain, heart, intestine, muscle, and liver using a rabbit monoclonal antibody (1:10,000, 40143-R019, Sino Biological). Viral antigen distribution was scored semi-quantitatively on a 0–3 scale, as previously described (Rockx et al., 2020; Brustolin et al., 2021; Vidal et al., 2022): 0 = absent, 1 = low, 2 = moderate, and 3 = high.

H&E-stained cervical region sections collected at 20, 40, and 60 dpi were digitized using a scanner (Aperio, Leica Biosystems) and analyzed blindly using Aperio ImageScope (v12.4.6.5003, Leica Biosystems). The cross-sectional area (CSA) of the cervical vagus nerve was determined from diameter measurements following established protocols (Ruiz-Casas et al., 2025). Briefly, the vagus nerve was identified based on its anatomical location, and diameters were measured perpendicularly to the orientation of the nerve fibers at a standardized level. Measurements were obtained from 12/14 mice at 20 dpi, 9/13 at 40 dpi, and 22/33 at 60 dpi.

### 2.10 Neutralizing antibody testing

Plasma neutralizing activity was assessed in duplicate using the cPass SARS-CoV-2 Neutralization Antibody Detection Kit (L00847, GenScript), following the manufacturer’s instructions. A SARS-CoV-2 neutralizing antibody calibrator (L00487, GenScript) was used to generate a standard curve for semi-quantitative analysis.

To prevent signal saturation and exceeding the assay’s upper detection limit (600 U/mL), plasma was diluted 1:300 (instead of the recommended 1:10), enabling discrimination of individual responses within the SARS-CoV-2 group.

Optical density (OD) was measured at 450 nm using a PowerWave XS microplate reader (BioTek). Neutralizing activity was calculated as percent inhibition: % inhibition = (1 − OD sample/OD negative control) × 100. Samples with < 30% inhibition were considered negative and assigned a value of 0 U/mL. Positive samples were quantified using the standard curve and expressed as U/mL.

### 2.11 Enzyme-linked immunospot (ELISpot) assay

SARS-CoV-2-specific T-cell responses at 60 dpi were assessed using an IFN*γ* ELISpot assay (3321-4APT-2, Mabtech). Cryopreserved splenocytes were rapidly thawed and resuspended in RPMI 1640 supplemented with 10% FBS, 1% glutamine, and 1% penicillin/streptomycin. Cells were plated at 3 × 10^5^ cells/well (100 μL) in 96-well plates and stimulated in triplicate with SARS-CoV-2 S protein (10 μg/mL; 40589-V08B1, Sino Biological), N protein (10 μg/mL; 40588-V08B, Sino Biological), or Concanavalin A (ConA, 7 μg/mL; L7647, Sigma-Aldrich™) as a positive control. Culture medium alone served as the negative control (background).

Following 24 h of incubation at 37 °C, plates were developed according to the manufacturer’s protocol. Spot-forming cells (SFC) were quantified using an AID iSpot Reader and AID ELISpot Software (version 8.0; Autoimmun Diagnostika GmbH). Assays were considered valid when ConA stimulation produced ≥ 100 spots and at least a 3-fold increase over unstimulated controls. Background correction was performed by subtracting counts from corresponding negative control wells.

### 2.12 Total protein and cytokine quantification

Cytokine concentrations were measured in lung, brain, and plasma samples collected at necropsy (10, 20, 40, and 60 dpi), as well as in longitudinal plasma samples (20 and 40 dpi) from animals euthanized at the study endpoint. Tissue cytokine levels were normalized to total protein content.

Lung and brain tissues were homogenized and centrifuged (16,000×*g*, 10 min, 4 °C). Supernatants were diluted 1:2 in PBS and adjusted to a final protein concentration of approximately 10 mg/mL. Total protein was quantified using the Pierce™ BCA Protein Assay Kit (23,227, Thermo Fisher Scientific) following the manufacturer’s instructions.

Cytokines, including CRP, CCL11, IFNγ, IL-1β, IL-10, IL-2, IL-4, IL-6, IP-10 (CXCL10), MIP-1α (CCL3), RANTES (CCL5), and TNFα, were quantified using multiplex Luminex assays (Procartaplex, Thermo Fisher Scientific) and expressed as pg/mg protein. Plasma samples were centrifuged (10,000×*g*, 10 min, 4 °C) prior to analysis. A 12-plex panel (MX9HMPF) was used for tissue samples, while plasma cytokines were measured using an 11-plex panel (MXPRNUE), with CRP measured separately using a single-plex assay (EPX1A-2604) at a 1:1000 dilution in Universal Assay Buffer.

All assays were run on a Luminex MagPix® system (Luminex Corporation) and analyzed using RBM Plate Viewer (v4.1.0.16979, Rules Based Medicine) and the ProcartaPlex Analysis App (Thermo Fisher Scientific).

### 2.13 Statistical analyses

Data distributions were assessed using the Shapiro–Wilk test for normality. Homogeneity of variances was evaluated using either the *F*-test or Brown–Forsythe test, as appropriate.

Survival analysis was performed using Kaplan–Meier curves, with group comparisons assessed by the Mantel–Cox (log-rank) test.

Clinical outcomes (clinical score, body weight, and temperature), behavioral measures, histopathological scores, and cytokine levels were analyzed using two-way ANOVA or mixed-effects models, as appropriate. Repeated measures across time points were included when applicable, followed by Šídák’s *post hoc* correction for multiple comparisons.

For behavioral Δ scores, inter-group variance was additionally assessed using the *F*-test with Bonferroni correction. Behavioral performance was further normalized using z-scores, calculated as previously described (Guilloux et al., 2011), with values <1.96 considered biologically relevant.

ELISpot data were analyzed using the Kruskal–Wallis test followed by Dunn’s multiple comparisons post hoc test.

Principal component analysis (PCA) was performed on log_2_ fold-change cytokine data. Samples were ordered in the heatmap based on PC1 and PC2 scores and loadings, enabling clustering of individuals and cytokines with similar expression profiles.

Associations between peak acute clinical severity and post-acute outcomes were evaluated using Pearson correlation analysis in animals euthanized at 60 dpi (*n* = 33). Analyses included lung and brain cytokine profiles, humoral and cellular immune responses, histopathological outcomes, and behavioral Δ scores. Correlations were additionally stratified by sex (males: G1, *n* = 4; G2, *n* = 10; females: G1, *n* = 4; G2, *n* = 15).

All statistical analyses were performed using GraphPad Prism v10.1.2, with significance set at *α* < 0.05.

## 3 Results

### 3.1 Consistent acute disease with reduced mortality following low-dose SARS-CoV-2 inoculation

In Study 1, disease severity increased in a dose-dependent manner. Survival rates were 100, 50, 25, and 0% following inoculation with 5 × 10^0^, 10^1^, 10^2^, and 10^3^ TCID_50_/animal, respectively (Supplementary Figure S2). Animals receiving ≥ 10^1^ TCID_50_ showed consistent body weight loss and detectable viral shedding in oropharyngeal swabs, whereas those inoculated with 0.5 × 10^1^ TCID_50_ maintained stable body weight and exhibited variable viral shedding (Supplementary Figure S2).

Based on these results, 10^1^ TCID_50_ was selected for the longitudinal assessment of long-term outcomes in Study 2 (Figure 1), as it consistently induced infection and measurable clinical signs while limiting mortality, thereby enabling assessment of both acute disease progression and potential post-acute outcomes.

Overall survival in Study 2 was 60% (47/78), with a non significant trend toward higher survival in females (68%, 27/40) compared with males (53%, 20/38) (Supplementary Figure S3). A total of 31 mice reached HEP criteria and were excluded from downstream analyses. Humane endpoints were reached between 5 and 12 dpi (5 dpi: n = 1; 6 dpi: n = 9; 7 dpi: n = 16; 8 dpi: n = 3; 9 dpi: n = 2; 10 dpi: n = 1; and 12 dpi: n = 1), and were defined by either a score of 3 in any individual clinical parameter (e.g., ≥20% body-weight loss) or a composite clinical score ≥ 10 (Supplementary Figure S3). Endpoint criteria were frequently accompanied by a marked decline in body temperature (Supplementary Figure S3).

Necropsy of animals reaching HEP between 5 and 7 dpi revealed consistent viral shedding in oropharyngeal swabs, nasal turbinate, and brain, with lower and more variable levels in heart, intestine, muscle, and liver (Supplementary Figure S3). Infectious virus was recovered from nasal turbinate, lung, and brain tissues (Supplementary Figure S3). SARS-CoV-2 N protein was low or absent in nasal turbinate, absent to moderate in lung, and moderate to high in brain (Supplementary Figure S3). Histopathological lesions were absent or mild in lung and mild to moderate in brain (Supplementary Figure S3), while no infectious virus, N protein, or lesions were detected in the remaining tissues examined.

Overall, the highest levels of viral gRNA (2.3 × 10^9^ copies/mg), infectious viral titers (mean: 10^6.4^ TCID_50_/mL), and N protein (score: 3) were consistently observed in the brain (Supplementary Figure S3).

Importantly, animals showing acute neuroinvasion and high viral burden did not survive the acute phase (Supplementary Figure S3) and were therefore excluded from the longitudinal cohort. Accordingly, the experimental design (Figure 1) includes only mice that survived the acute phase with a non-lethal disease course. In these animals, no viral RNA, infectious virus, or nucleocapsid antigen was detected in the brain at post-acute time points, and no histopathological evidence of encephalitis was observed. Thus, all longitudinal outcomes reflect post-acute sequelae in the absence of ongoing CNS viral infection.

### 3.2 Time- and sex-dependent clinical manifestations throughout the study period

**Figure 2:**
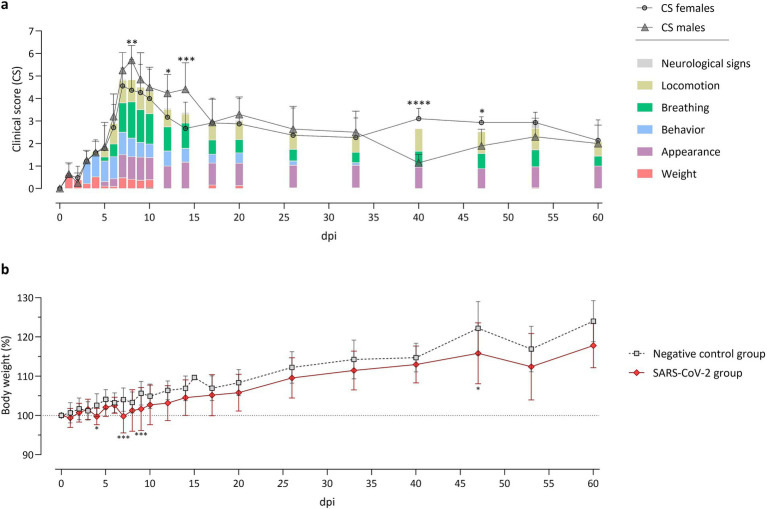
Clinical scores and body weight changes over 60 days post-inoculation (dpi). **(a)** Mean clinical scores (0–3) over time in SARS-CoV-2-inoculated mice, categorized as neurological signs (gray), locomotion (yellow), breathing (green), behavior (blue), appearance (purple), and body weight (red). Total clinical scores are shown separately for males (triangles) and females (circles). Higher scores indicate greater disease severity. **(b)** Mean body weight variation relative to baseline (%) in negative controls (G1; gray squares) and SARS-CoV-2-inoculated mice (G2; red rhomboids). Data are presented as means ± standard deviations. Group sizes are as follows: 0–10 dpi, G1 *n* = 26 and G2 *n* = 47; 10–20 dpi, G1 *n* = 20 and G2 *n* = 41; 20–40 dpi, G1 *n* = 16 and G2 *n* = 33; 40–60 dpi, G1 *n* = 10 and G2 *n* = 25. Statistical significance was assessed using two-way repeated measures ANOVA followed by Šídák’s multiple comparisons test (*p < 0.05 and ***p < 0.001).

Clinical scores peaked at 7–8 dpi (mean ± standard deviation: 5 ± 1), reflecting abnormalities in locomotion, breathing, behavior, appearance, and body weight (Figure 2a). During the acute phase, males exhibited greater disease severity, showing significantly higher clinical scores than females at 8, 12, and 14 dpi, whereas females did not differ significantly from controls. In the post-acute phase, impairments in locomotion, breathing, and appearance persisted up to 60 dpi. Notably, the sex-related pattern reversed, with females displaying significantly higher clinical scores than males at 40 and 47 dpi (Figure 2a).

Body weight was also significantly reduced in the SARS-CoV-2 group compared with controls at 4, 7, and 9 dpi (Figure 2b). Marked inter-individual variability was observed in the minimum body weight relative to baseline, ranging from 83 to 100% of baseline. This variability was largely sex-dependent, as SARS-CoV-2-inoculated males showed significant body weight loss compared with male controls at 4, 7–9, 12, and 14 dpi, whereas females did not differ significantly from controls. During the post-acute phase, a significant reduction in body weight was still observed at 47 dpi but no sex-specific differences were detected at this stage (Figure 2b).

Subcutaneous temperature remained similar between surviving mice and control groups throughout the study (Supplementary Figure S4).

### 3.3 Time- and sex-dependent behavioral impairments following SARS-CoV-2 infection

Behavioral performance was assessed longitudinally using the BT, NBT, OFT, and EZMT.

At the group level, no significant differences in *Δ* scores were observed between SARS-CoV-2-inoculated and control mice across behavioral tests or time points (Supplementary Figure S5). In the BT, all mice completely emptied the tube within 30 min at each assessment, resulting in Δ scores of 0 throughout the study period. Likewise, NBT performance remained stable across groups and time points (Supplementary Figure S5). In the OFT, total distance moved did not differ between groups, indicating preserved locomotor activity (Supplementary Figure S5). However, marked inter-individual variability was observed in the center-to-total distance ratio and time spent in the center (Supplementary Figure S5), with greater variance in SARS-CoV-2-inoculated mice compared with controls at 21 dpi. Similarly, in the EZMT, variability in both the frequency of open-zone entries and time spent in the open zones increased in inoculated mice, with variance in open-zone entry frequency elevated at 42 dpi (Supplementary Figure S5).

**Figure 3:**
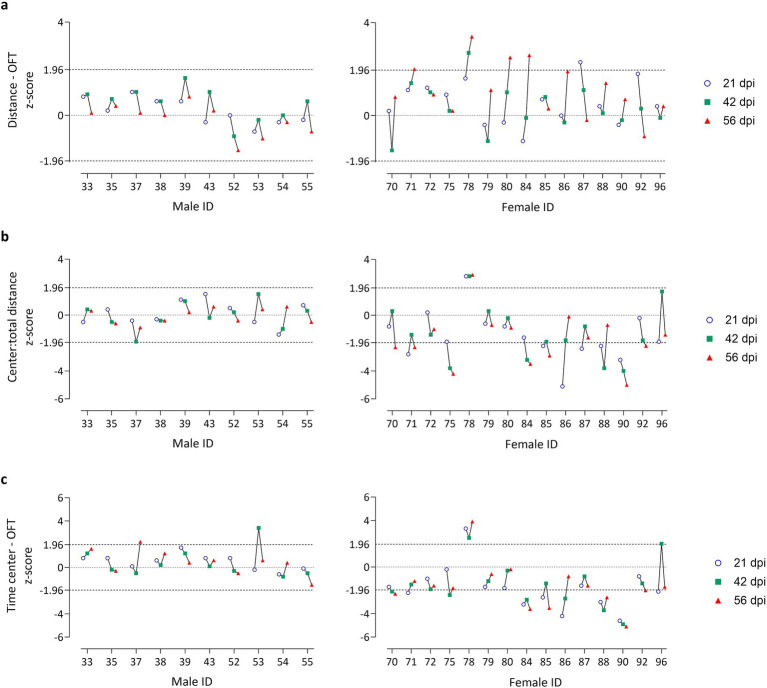
Open field test (OFT) performance following SARS-CoV-2 inoculation. Individual z-scores for SARS-CoV-2-inoculated animals are shown for **(a)** total distance moved, **(b)** center-to-total distance, and **(c)** time spent in the center at 21 (blue circles), 42 (green squares), and 59 (red triangles) days post-inoculation (dpi). Left panels correspond to males (ID 33–55) and right panels to females (ID 70–96). Z-scores were calculated from delta values (post-challenge minus pre-challenge value) normalized to the mean and standard deviation of the negative control group. The dotted line indicates a z-score of 0, and dashed lines indicate the ±1.96 threshold for deviation from controls.

**Figure 4:**
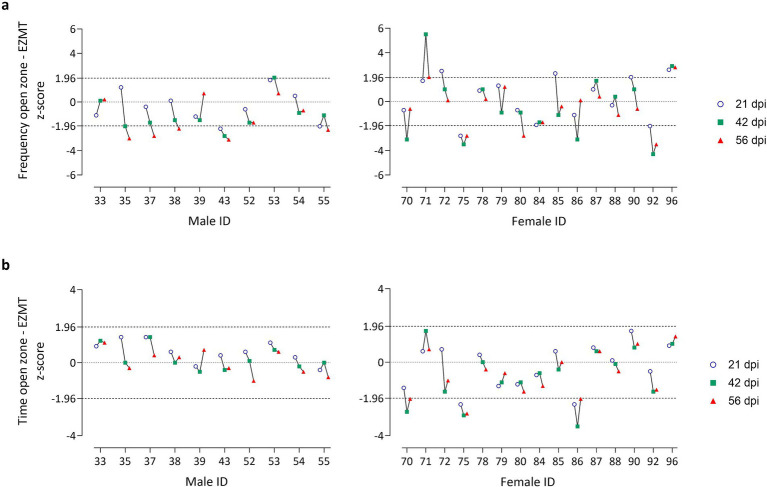
Elevated zero maze test (EZMT) performance following SARS-CoV-2 inoculation. Individual z-scores for SARS-CoV-2-inoculated animals are shown for **(a)** frequency of entries into the open zones, and **(b)** time spent in the open zones at 21 (blue circles), 42 (green squares), and 59 (red triangles) days post-inoculation (dpi). Left panels correspond to males (ID 33–55) and right panels to females (ID 70–96). Z-scores were calculated from delta values (post-challenge minus pre-challenge value) normalized to the mean and standard deviation of the negative control group. The dotted line indicates a z-score of 0, and dashed lines indicate the ±1.96 threshold for statistical significance.

Given the marked inter-individual heterogeneity observed in OFT and EZMT performance, subsequent analyses focused on individual-level z-scores (Figures 3, 4). These scores quantify how far each Δ value deviates from the mean Δ score of the negative control group, expressed in units of standard deviation. In the OFT, total distance moved remained unchanged in both males (Figure 3a, left panel) and females (Figure 3a, right panel). The center-to-total distance ratio remained unchanged in males (Figure 3b, left panel) but was reduced (z < −1.96) in a subset of females (n = 10/15; ID 70, 71, 75, 84–88, 90, 92; Figure 3b, right panel). Similarly, time spent in the center was unchanged in males (Figure 3c, left panel), whereas it was reduced in a largely overlapping subset of females (n = 10/15, ID 70, 71, 75, 84–86, 88, 90, 92, 96; Figure 3c, right panel). In the EZMT, the frequency of entries into the open zones was reduced in subsets of both males (n = 5/10; ID 35, 37, 38, 43, 55; Figure 4a, left panel) and females (n = 5/15; ID 70, 75, 80, 86, 92; Figure 4a, right panel). Time spent in the open zones remained unchanged in males (Figure 4b, left panel) but was reduced in a subset of females (n = 3/15; ID 70, 75, 86) (Figure 4b, right panel).

A comprehensive analysis of the most affected parameters (OFT center-to-total distance ratio, OFT time spent in the center, and EZMT frequency of entries into the open zones) revealed distinct time- and sex-dependent patterns. The number of animals exhibiting behavioral impairments varied across time points, affecting 12/25 at 21 dpi, 9/25 at 42 dpi, and 14/25 at 56 dpi (Supplementary Figure S6). The nature of behavioral impairments also changed over time. At 21 dpi, deficits were primarily detected in OFT-derived measures (Supplementary Figure S6a). By 42 dpi, impairments became more prominent in anxiety-related behaviors, including reduced time spent in the center and decreased frequency of entries into the open zones (Supplementary Figure S6b). At 56 dpi, deficits were observed across all assessed parameters (Supplementary Figure S6c). Sex-specific patterns also emerged, with females (ID 70–96) consistently showing lower z-scores, predominantly in OFT-derived measures, whereas males (ID 35–55) displayed alterations mainly in EZMT performance (Supplementary Figure S6).

### 3.4 Persistence of SARS-CoV-2 gRNA in respiratory tissues and pulmonary lesions

**Figure 5:**
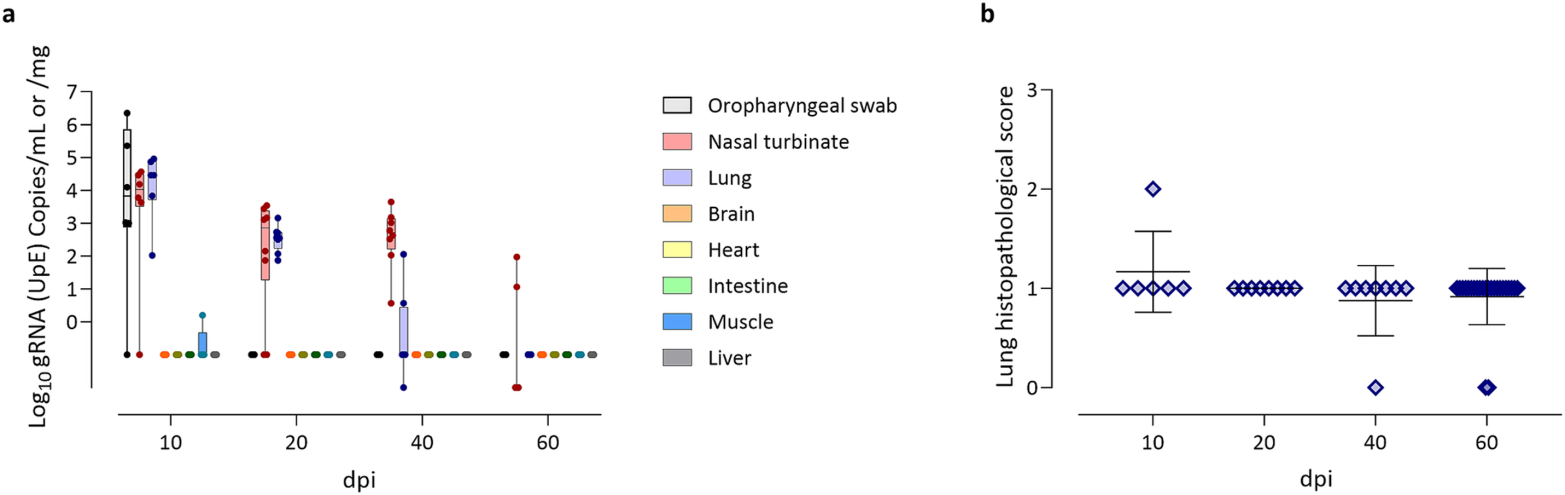
Detection of SARS-CoV-2 genomic RNA (gRNA) and lung lesions over time. **(a)** SARS-CoV-2 gRNA levels detected by RT-qPCR targeting the envelope protein gene (UpE) in oropharyngeal swabs (light gray; log_10_ gRNA copies/mL) and tissues, including nasal turbinate (red), lung (blue), brain (orange), heart (yellow), intestine (green), muscle (blue), and liver (dark gray), expressed as log_10_ gRNA copies/mg. **(b)** Semi-quantitative histopathological scores of lung inflammatory lesions (0 = absent, 1 = mild, 2 = moderate), shown as individual values with means ± standard deviations. SARS-CoV-2-inoculated group sizes were *n* = 6 at 10 dpi, *n* = 8 at 20 dpi, *n* = 8 at 40 dpi, and *n* = 25 at 60 dpi.

SARS-CoV-2 gRNA was detected in oropharyngeal swabs, nasal turbinate, and lung tissues from all necropsied mice at 10 dpi (Figure 5a). Viral gRNA remained detectable in the lung until 20 dpi and in the nasal turbinate until 40 dpi. By 60 dpi, gRNA remained detectable only in the nasal turbinate of 2/10 mice. In contrast, gRNA was undetectable in all extra-respiratory tissues examined, including brain, heart, intestine, muscle, and liver, at all time points (Figure 5a).

Histopathological analysis revealed persistent, predominantly mild pulmonary lesions throughout the study. At 10 dpi, lungs exhibited multifocal broncho-interstitial pneumonia of mild (*n* = 5) to moderate (*n* = 1) severity. By 20 dpi, lesions had evolved predominantly into mild peribronchial lymphoid hyperplasia (*n* = 8), which persisted at 40 dpi (*n* = 7/8) and 60 dpi (*n* = 22/24) (Figure 5b and Supplementary Figure S7). No histopathological abnormalities were observed in nasal turbinate, brain, heart, intestine, muscle, or liver tissues.

Across all tissues and time points (≥ 10 dpi), infectious virus remained below the assay limit of quantification (< 10^1.8^ TCID_50_/mL), and SARS-CoV-2 N protein was undetectable by IHC, indicating minimal or absent productive viral replication.

### 3.5 Long-term reduction in vagus nerve cross-sectional area following SARS-CoV-2 inoculation

**Figure 6:**
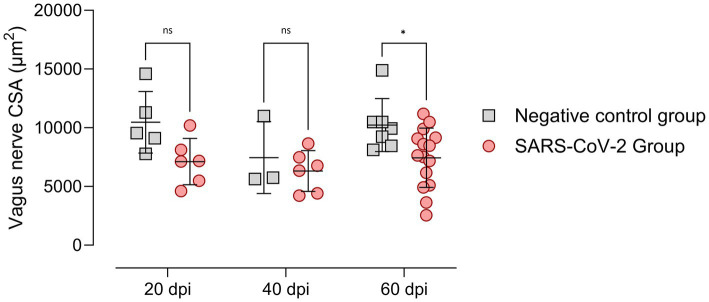
Vagus nerve cross-sectional area (CSA) at multiple post-acute time points. Cervical vagus nerve CSA (μm^2^) is shown as individual data points for negative controls (G1; grey squares) and SARS-CoV-2-inoculated mice (G2; red circles) at 20, 40, and 60 days post-inoculation (dpi). Group sizes were: 20 dpi, G1 *n* = 5 and G2 *n* = 6; at 40 dpi, G1 *n* = 4 and G2 *n* = 6; and at 60 dpi, G1 *n* = 7 and G2 *n* = 16. Data are presented as means ± standard deviations. Statistical significance was assessed using two-way repeated-measures ANOVA followed by Šídák’s multiple comparisons test (*p < 0.05).

The mean CSA of the cervical vagus nerve was reduced was reduced at 20, 40, and 60 dpi in SARS-CoV-2-inoculated mice compared with controls, reaching statistical significance at 60 dpi (Figure 6).

### 3.6 Time- and sex-dependent cytokine dynamics in lung and brain after SARS-CoV-2 inoculation

**Figure 7:**
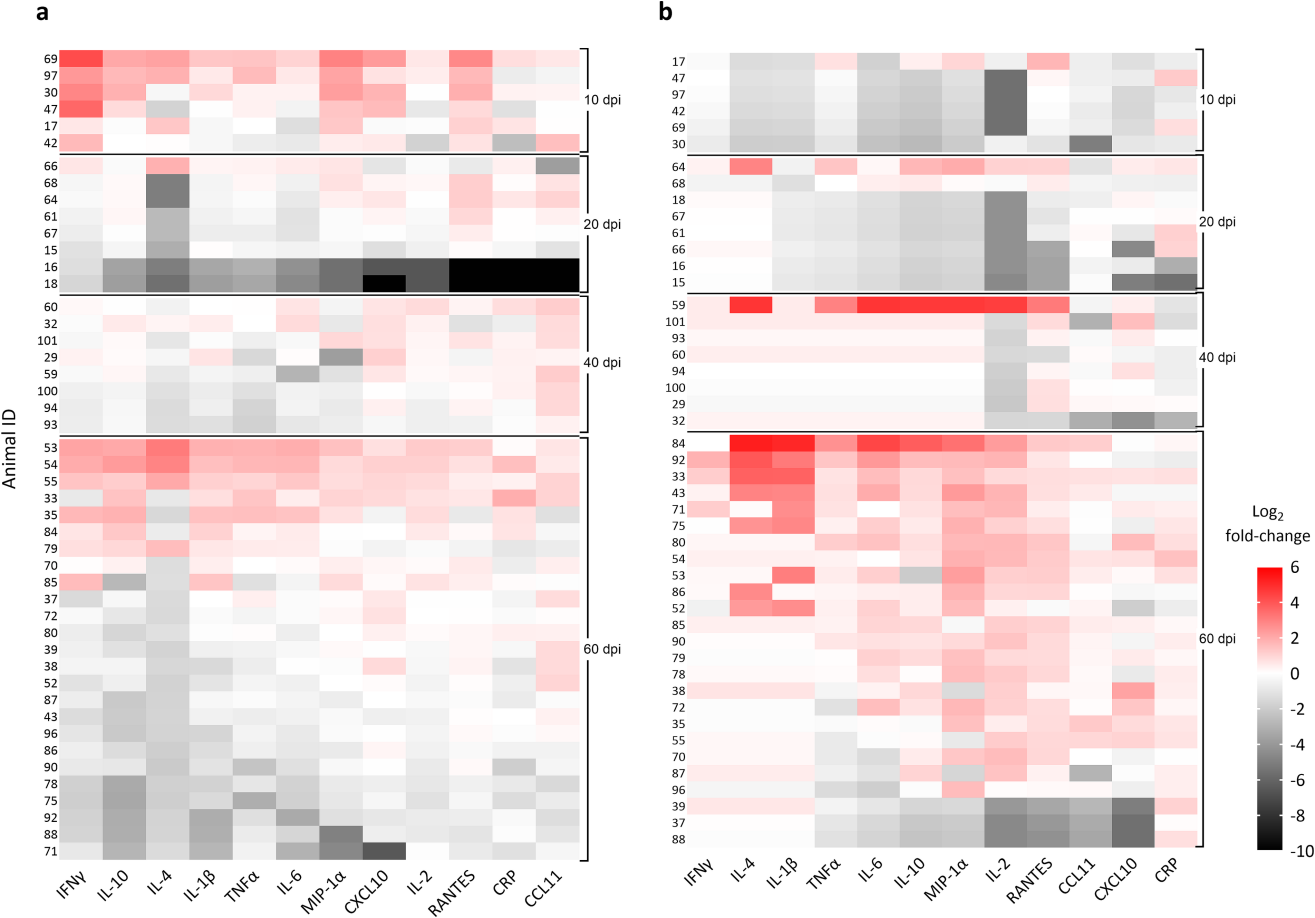
Lung and brain cytokine profiles following SARS-CoV-2 inoculation. Heatmaps showing log₂ fold changes in cytokine concentrations in the **(a)** lung and **(b)** brain at 10, 20, 40, and 60 dpi. Cytokine levels (IL-10, MIP-1α, IL-1β, TNFα, CRP, RANTES, CXCL10, IFNγ, IL-2, IL-4, and CCL11) were normalized to total protein (pg/mg) and calculated relative to the mean values of negative controls. Red indicates cytokine upregulation and black indicates downregulation relative to controls. Rows (individual animals) and columns (cytokines) were ordered according to principal component (PC) 1 and PC2 scores and loadings derived from principal component analysis (PCA) of the log₂ fold-change dataset, grouping samples and cytokines with similar expression profiles adjacent to one another.

In the lung, cytokine profiles exhibited marked temporal and sex-dependent modulation following SARS-CoV-2 inoculation. At 10 dpi, most animals displayed robust upregulation of pro-inflammatory cytokines, including INFγ, IL-10, TNFα, MIP-1α, CXCL10, and RANTES (Figure 7a), with significant group-level increases (Supplementary Figure S8). At 20 dpi, multiple cytokines became strongly downregulated (Figure 7a), although TNFα remained significantly elevated in inoculated animals compared with controls (Supplementary Figure S8). By 40 dpi, lung cytokine levels had largely returned to baseline, with only modest increases in CXCL10 and CCL11 observed in some animals (Figure 7a), and no significant differences detected at the group level (Supplementary Figure S8). At 60 dpi, lung cytokine responses became highly heterogeneous. This variability was driven primarily by a subset of males (ID 53, 54, 55, 33, and 35) showing late upregulation of multiple pro-inflammatory cytokines and IL-4 (Figure 7a). Correspondingly, significant group-level increases in INFγ, IL-1β, TNFα, CXCL10, IL-2, and CCL11 were observed relative to controls (Supplementary Figure S8). Overall, males exhibited higher cytokine concentrations than females and controls, whereas females showed a significant increase only in TNFα.

In the brain, cytokine modulation was more variable over time. At 10 dpi, most animals showed baseline or reduced cytokine levels (Figure 7b), with significant decreases in IL-2, CCL11, and CXCL10 compared with controls (Supplementary Figure S9). At 20 and 40 dpi, cytokine levels largely approached baseline, although sporadic cytokine upregulation (> 2 log_2_ fold-change) was observed in two males (ID 59, 64; Figure 7b). At 60 dpi, pronounced heterogeneity emerged in brain cytokine profiles, with multiple animals (ID 84, 92, 33, 43, 71, 75, 80, 54, 53, 86, and 52) displaying broad cytokine upregulation (Figure 7b). This was associated with significant group-level increases in MIP-1α, IL-2, and RANTES compared with controls (Supplementary Figure S9).

In contrast, plasma cytokine concentrations remained unchanged throughout the study.

### 3.7 Sustained adaptive immune responses following SARS-CoV-2 inoculation

Neutralizing antibodies were first detected at 10 dpi in 2/6 SARS-CoV-2-inoculated mice. The remaining samples showed < 30% inhibition at a 1:300 dilution and were therefore considered negative. Neutralizing antibody levels increased markedly by 20 dpi (mean: 9,878 U/mL) and plateaued by 40 dpi (mean: 10,314 U/mL). At 60 dpi, antibody titers were highly variable (range: 1,921–17,466 U/mL), indicating substantial heterogeneity in the magnitude and persistence of the humoral response (Supplementary Figure S10).

IFNγ ELISpot responses were background-corrected using unstimulated wells (mean background: 25 SFC/10^6^ cells). At 60 dpi, splenocytes generally exhibited stronger responses following stimulation with SARS-CoV-2 S protein than with N protein. Females (ID 70–96) exhibited the highest responses (mean SFC/10^6^; S: 22; N: 11.9), followed by males (ID 33–55; mean SFC/10^6^; S: 8.3; N: 6.4), whereas negative controls showed minimal responses. However, due to high inter-individual variability, no statistically significant group differences were detected (Supplementary Figure S10).

### 3.8 Acute phase disease severity predicted long-term immune and neurocognitive outcomes in a sex-dependent manner

Correlation analyses were performed between peak acute clinical scores and post-acute outcomes in animals euthanized at 60 dpi (*n* = 33), with significant associations summarized in Supplementary Table S2.

In males, greater acute clinical severity was positively associated with higher neutralizing antibody levels and elevated lung pro-inflammatory cytokine concentrations at 60 dpi, including CCL11, IL-6, IFNγ, and TNFα. Acute disease severity also correlated negatively with vagus nerve CSA at 60 dpi.

In females, greater acute clinical severity was positively associated with neutralizing antibody levels and brain CRP concentrations at 60 dpi. In addition, higher acute severity correlated with impaired OFT performance, including reduced center-to-total distance ratio at 21 and 42 dpi, as well as decreased time spent in the center at 21, 42, and 56 dpi.

## 4 Discussion

PCC encompasses a broad spectrum of multisystemic symptoms that can persist for years, substantially impairing quality of life in millions of individuals worldwide. Neurological and neuropsychiatric manifestations are among the most prevalent and disabling features, affecting cognition, emotional wellbeing, and daily functioning. Despite their clinical relevance, the biological mechanisms driving neuro-PCC remain poorly understood, highlighting critical gaps in diagnosis and treatment strategies (Ely et al., 2024; Moen et al., 2025). Together, the findings presented here demonstrate that, while ~40% of animals developed severe disease with evidence of neuroinvasion after SARS-CoV-2 challenge and were therefore excluded from the longitudinal analyses, the remaining ~60% survived the acute phase with a mild-to-moderate disease course. In these animals, low-dose viral inoculation induced persistent, sex-dependent neurological and immunological alterations in K18-hACE2 mice, despite the absence of detectable viral replication in the brain during the post-acute phase.

K18-hACE2 mice are widely used in SARS-CoV-2 research due to their high susceptibility to ancestral SARS-CoV-2 strain and the extensive availability of genetic and immunological tools (Usai et al., 2023). However, severe neuroinvasion and high mortality have limited their utility for long-term studies of PCC (Vidal et al., 2022). Here, we optimized this model using a low-dose inoculum of the SARS-CoV-2 B.1 strain (D614G), enabling survival and longitudinal assessment up to 60 dpi, roughly equivalent to several human years (Dutta and Sengupta, 2019). Importantly, this approach preserved measurable acute disease while minimizing mortality, thereby generating a clinically relevant framework to investigate long-term sequelae associated with early SARS-CoV-2 variants, which have been linked to increased PCC risk in humans (Lok et al., 2024).

A major finding of this study was the emergence of sex-dependent disease trajectories during both acute and post-acute phases. Males developed more severe acute disease, consistent with human COVID-19 sex-bias and previous mouse studies (Jiang et al., 2020; Peckham et al., 2020; Aiolfi et al., 2022; Onodera et al., 2023). In contrast, females showed higher post-acute clinical scores and more pronounced behavioral impairments, aligning with epidemiological studies indicating that PCC disproportionately affects women (Shah et al., 2025). These divergent trajectories likely reflect sex-specific immunobiology, including X-linked immune regulation and estrogen-mediated effects on antiviral responses and ACE2 expression, which may enhance acute resistance in females (Gemmati et al., 2020; Rehman et al., 2021; Onodera et al., 2023). However, enhanced immune activation in females may also predispose to prolonged neuroimmune dysregulation and chronic inflammatory sequelae (Hamlin et al., 2024; Maham and Yoon, 2024). Behavioral analyses indicated persistent and heterogeneous neurocognitive alterations following SARS-CoV-2 infection. Although group-level analyses failed to detect significant differences, individual-level z-score analyses identified subsets of animals with sustained impairments in exploratory and anxiety-related behaviors. Deficits were already evident at 21 dpi, partially improved by 42 dpi, and became more heterogeneous again by 56 dpi. Females predominantly exhibited OFT-related impairments, whereas males showed alterations mainly in EZMT performance. These findings closely resemble the relapsing–remitting and heterogeneous trajectories described in human neuro-PCC, where symptom severity and clinical presentation may vary substantially among individuals and between sexes (Ely et al., 2024; Gorenshtein et al., 2024; Choudhury et al., 2025; Toepffer et al., 2025). Importantly, the absence of locomotor deficits suggests that the observed behavioral changes were not secondary to generalized physical impairment but instead reflected selective alterations in anxiety-related or neurocognitive domains.

Persistent pulmonary pathology and immune dysregulation were also evident throughout the post-acute phase. Despite clearance of detectable infectious virus, mild pulmonary lesions and dyspnea persisted up to 60 dpi, recapitulating key clinical features of human PCC (Ravaglia et al., 2022; Patton et al., 2024). Unlike previous murine studies reporting mainly fibrotic repair (Dinnon et al., 2022), our model showed sustained peribronchial lymphoid hyperplasia and sustained cytokine dysregulation, suggesting incomplete resolution of inflammation (Calvanese et al., 2024). Late pulmonary cytokine upregulation at 60 dpi reflected activation of innate inflammatory pathways (IL-1β, TNFα) (Parameswaran and Patial, 2010; Ford et al., 2025), Th1-associated responses (TNFα, IFNγ, CXCL10, IL-2), and Th2-associated signaling (CCL11) (Odaka et al., 2007; Zeng et al., 2022). Males displayed stronger pulmonary cytokine responses than females, consistent with clinical observations linking male sex to more severe pulmonary sequelae following COVID-19 (Patton et al., 2024).

Notably, elevated CCL11 was detected in several males exhibiting behavioral deficits (3 out of 5), supporting a mechanistic link between chronic pulmonary inflammation and neurological sequelae (Fernández-Castañeda et al., 2022). CCL11 has previously been associated with lung injury, impaired neurogenesis, cognitive decline, and neuro-PCC (Huaux et al., 2005; Villeda et al., 2011; Fernández-Castañeda et al., 2022). Although no viral replication or structural lesions were detected in the brain of surviving animals, cytokine analyses revealed persistent neuroinflammatory signatures at 60 dpi. Elevated IL-2, RANTES, and MIP-1α suggest sustained T-cell and myeloid activation within the CNS, pathways associated with synaptic dysfunction, blood–brain barrier disruption, and chronic neurological disorders (Terao et al., 2008; Marciniak et al., 2015; Wylezinski and Hawiger, 2016; Zhang et al., 2025). These findings support the hypothesis that post-acute neuroinflammation can persist independently of active viral replication, which may be driven by residual viral antigens, systemic inflammation, or altered blood–brain barrier permeability. Although these mechanisms were not directly investigated in the present study, they have been proposed as key contributors to chronic neurological manifestations in both human neuro-PCC and experimental models (Rhea et al., 2021; Etter et al., 2022; Fontes-Dantas et al., 2023; Rong et al., 2024; Popa et al., 2025). Alterations in the vagus nerve further support the presence of sustained autonomic dysfunction after SARS-CoV-2 infection. Vagus nerve abnormalities have been reported in PCC patients and are linked to dysautonomia, impaired anti-inflammatory signaling, and cognitive dysfunction (Camici et al., 2024; Khan et al., 2024; Lladós et al., 2024). In our model, SARS-CoV-2-inoculated mice exhibited a significant reduction in vagus nerve CSA at 60 dpi. This finding is consistent with observations in patients with PCC, in whom reduced vagus nerve CSA has been reported and proposed to reflect underlying structural changes, including possible atrophy or degeneration (Papadopoulou et al., 2023). These findings suggest that peripheral autonomic pathways may contribute to long-term neurological sequelae independently of direct viral neuroinvasion. Severity of clinical manifestations during the acute phase emerged as an important predictor of long-term outcomes. Peak clinical scores correlated with sustained neutralizing antibody responses and reduced vagus nerve CSA at 60 dpi. In males, acute severity additionally correlated with persistent pulmonary inflammation, whereas in females it was associated with OFT impairments, including reduced exploratory behavior and increased anxiety-like responses. These sex-dependent associations mirror human studies in which severe COVID-19 increases the risk of persistent pulmonary dysfunction and neurocognitive sequelae, with pulmonary complications more prevalent in males and cognitive impairment more common in females (Brandstetter Figueroa et al., 2025; Moen et al., 2025; Verma et al., 2025). The marked heterogeneity observed among infected mice highlights the existence of distinct post-acute phenotypic subgroups. Individual-level analyses identified animals exhibiting predominantly neurobehavioural impairment (ID 35, 55, 84, and 92), others characterized mainly by vagus nerve CSA reduction (ID 70), and some displaying combined behavioral, inflammatory, and vagus nerve alterations (ID 85). These patterns resemble the clinical heterogeneity observed in human PCC, where multiple overlapping endotypes have been proposed (Ozonoff et al., 2024; Peluso and Deeks, 2024). Importantly, these findings emphasize the value of individualized analyses in preclinical PCC research, as group-level comparisons alone may obscure biologically relevant subpopulations.

Comparison with our previous work in golden Syrian hamsters (Ruiz-Casas et al., 2025) reveals both shared and model-specific features of post-acute disease. In both species, we observed sex-dependent neurobehavioral alterations together with persistent cytokine dysregulation at 60 dpi, suggesting that certain long-term consequences of SARS-CoV-2 infection are conserved across models. However, notable differences were also evident. In hamsters, long-term outcomes were characterized by persistent detection of viral gRNA in the absence of overt sustained pathological alterations in tissues, suggesting that residual viral material may contribute to chronic phenotypes in this model. In contrast, K18-hACE2 mice exhibited persistent clinical alterations, prolonged pulmonary pathology, and reduced vagus nerve CSA despite minimal or undetectable viral persistence at late time points. This pattern suggests that long-term sequelae in K18-hACE2 mice are less likely driven by ongoing viral presence and may instead reflect maladaptive or sustained host responses following acute infection. Additionally, the mouse study included a larger number of animals reaching the latest experimental time points, increasing the capacity to detect persistent alterations during the post-acute phase. This difference in study design may have further contributed to the more robust identification of long-term pathological findings in K18-hACE2 mice compared with hamsters. Taken together, these findings support the notion that distinct, and potentially complementary, mechanisms underlie post-acute outcomes across models, with a stronger contribution of viral persistence-related processes in hamsters and a more prominent role of sustained immune-driven alterations in K18-hACE2 mice.

While the present study provides important insights into the long-term neurological consequences of SARS-CoV-2 infection in K18-hACE2 mice, several limitations should be considered. As with all experimental models, this system captures only part of the complex and multifactorial nature of PCC in humans. Although conserved features between rodent and primate brains support the use of mouse models to investigate neuroimmune mechanisms relevant to human disease (Hodge et al., 2019; Kim et al., 2023; Beauchamp et al., 2022), important anatomical and functional differences remain, particularly in cortical organization, prefrontal cortex complexity, and long-range connectivity, which may limit direct translation of PCC-related behavioral phenotypes (Ventura-Antunes et al., 2013; Laubach et al., 2018; Ma et al., 2022). Accordingly, our behavioral findings should be interpreted as reflecting PCC-like neurobiological signatures rather than direct equivalents of human cognitive or neuropsychiatric symptoms. In addition, although the behavioral battery of tests used here allowed the detection of persistent neurobehavioral alterations, it primarily assessed emotionality and locomotion-related domains and does not fully capture key clinical features of PCC such as brain fog. Future studies incorporating dedicated paradigms assessing memory and cognitive flexibility will be important to better model the broader neurological spectrum of PCC. Likewise, while we observed reduced vagus nerve CSA, we did not perform detailed histopathological or functional analyses to determine whether this reflects demyelination, axonal injury, or other structural changes, which warrants further investigation. Furthermore, acute-phase mortality reduced the number of animals available for long-term follow-up.

Despite these limitations and inherent challenges in cross-species translation (including differences in lifespan, immune responses, viral kinetics, genetic background, and aging), the model provides a useful platform to investigate mechanisms underlying neuro-PCC and to evaluate potential therapeutic strategies targeting chronic inflammation, autonomic dysfunction, and neuroimmune signaling. Overall, our optimized K18-hACE2 mouse model recapitulates selected features of human PCC, encompassing persistent behavioral abnormalities, sustained pulmonary and neural inflammation, ongoing pulmonary pathology, and reduced vagus nerve CSA, in the absence of detectable viral replication in the brain. Notably, female sex and acute clinical severity emerged as major predictors of long-term outcomes, consistent with observations in PCC patients (Ely et al., 2024; Guillén-Teruel et al., 2025; Moen et al., 2025). Although this study does not capture all aspects of human PCC, it provides a meaningful experimental framework for studying post-acute SARS-CoV-2 sequelae and their underlying mechanisms.

## Data Availability

The original contributions presented in the study are included in the article/Supplementary material, further inquiries can be directed to the corresponding authors.
