# Supplementary material for: Persistent neurological and behavioral alterations after SARS-CoV-2 infection in an optimized K18-hACE2 mouse model
Source: Front Microbiol. 2026 Jun 26;17:1871084. doi: 10.3389/fmicb.2026.1871084 (PMC13350038; doi:10.3389/fmicb.2026.1871084)
Supplement: Supplementary file 1 [file Table_1.docx]

Supplementary Material

# Supplementary Figures and Tables

## Supplementary Figures


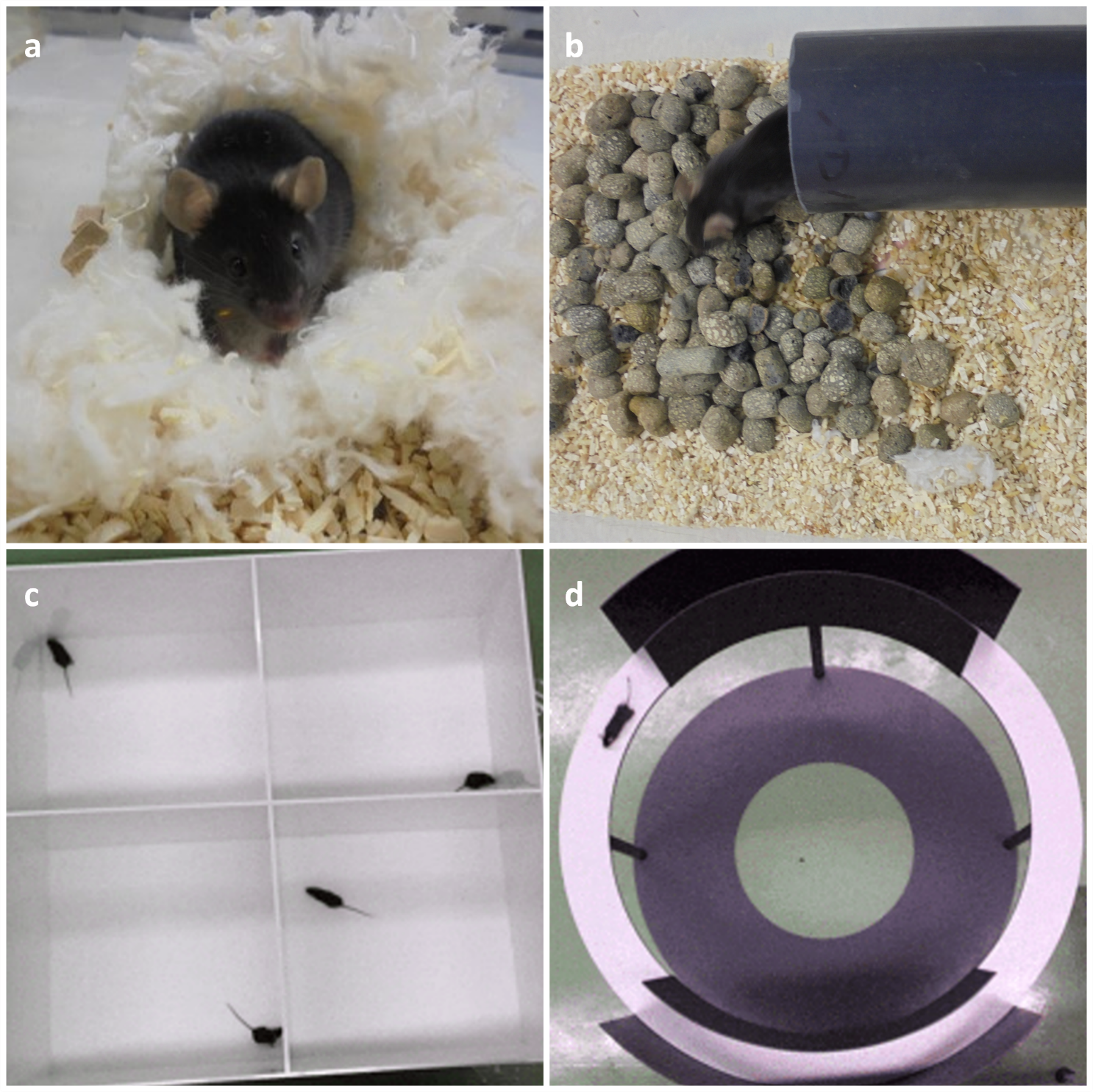


**Supplementary Figure S1.** **Behavioral tests used to assess neurocognitive performance. (a)** Nest-building test, **(b)** burrowing test, **(c)** open field test, and **(d)** elevated zero maze test.


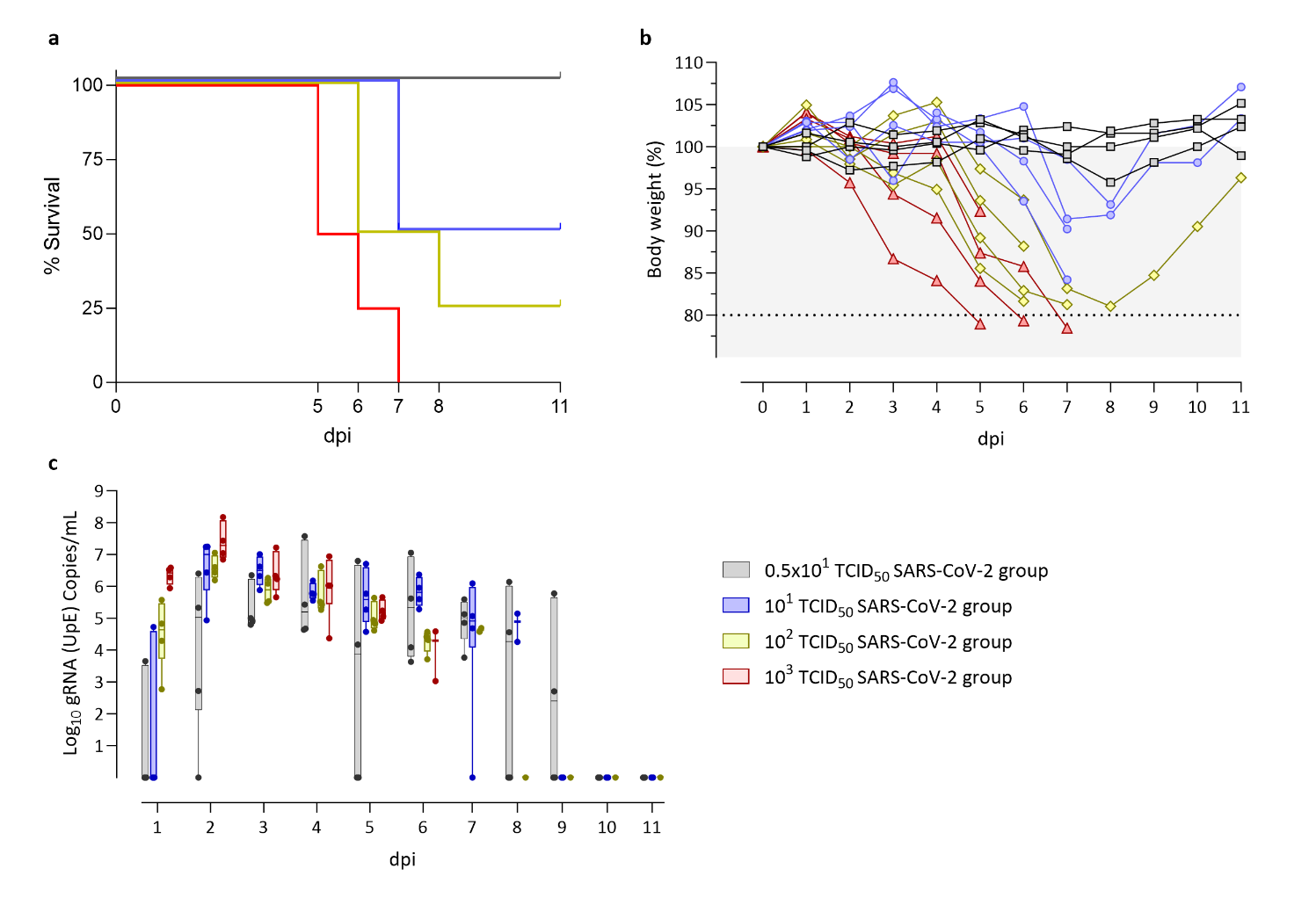


**Supplementary Figure S2.** **Dose-dependent clinical and virological responses for 11 days post-SARS-CoV-2 inoculation (dpi). (a)** Survivability (%), **(b)** body weight change (%), and **(c)** viral genomic RNA gRNA) of the upstream envelope gene (UpE) in oropharyngeal swabs (log_10_ copies/mL) in mice inoculated with 0.5 × 10^1^ (gray), 10^1^ (blue), 10^2^ (yellow), or 10^3^ (red) TCID_50_ per mouse (n = 4 per group).


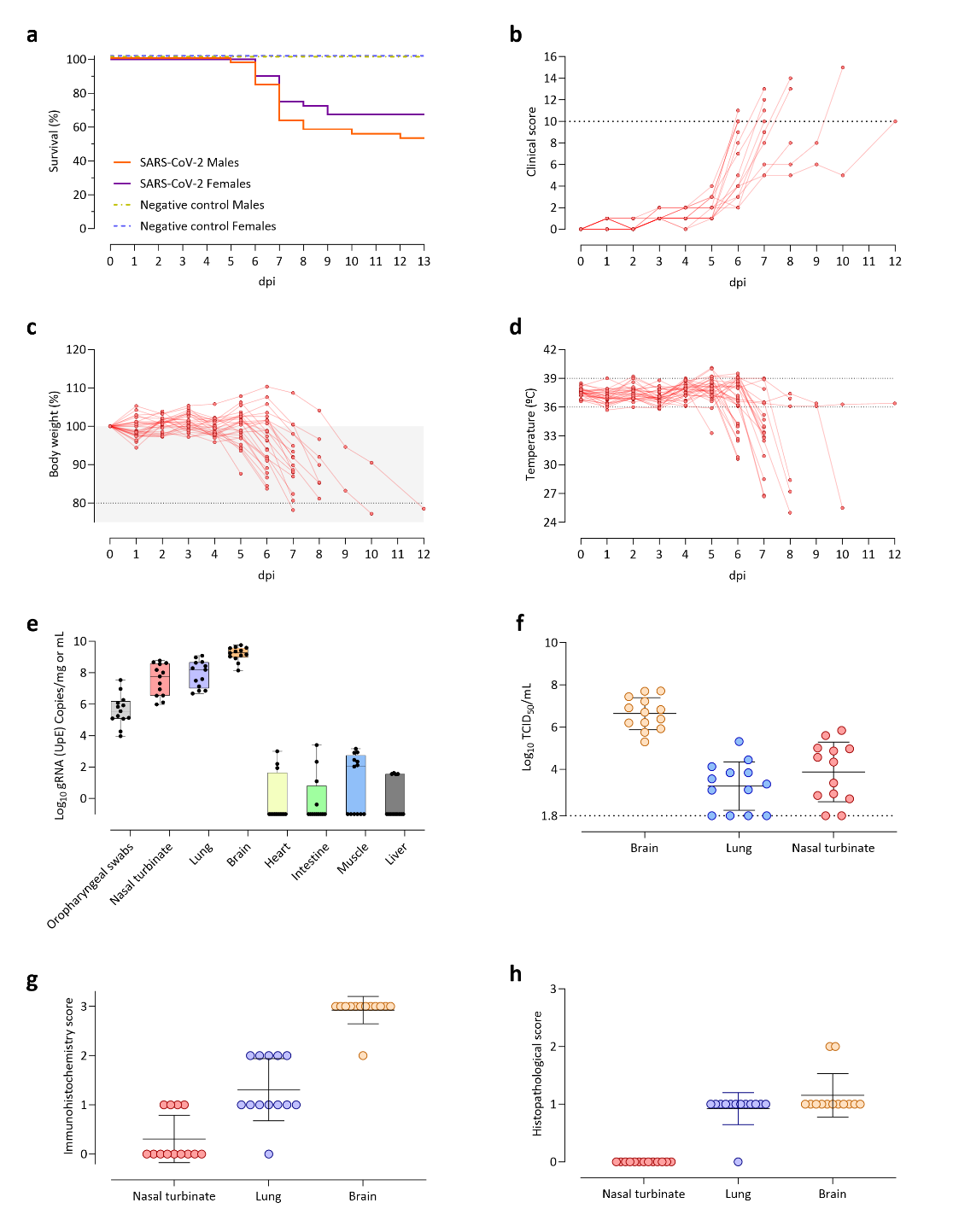


**Supplementary Figure S3.** **Clinical, virological, and histopathological outcomes in mice succumbing to 10^1^ TCID_50_ SARS-CoV-2 inoculation. (a)** Survivability (%) of SARS-CoV-2 inoculated males (orange, solid) and females (purple, solid), and negative control males (green, dashed) and females (blue, dashed). **(b)** Total clinical scores; the dotted line indicates the humane endpoint (HEP; total clinical score ≥ 10). **(c)** Body weight (% of baseline); the dotted line marks the HEP threshold (80%). **(d)** Subcutaneous temperature (°C); dotted lines indicate the normal physiological range (36-39 °C). **(e)** Genomic RNA (gRNA) copies of the upstream envelope gene (UpE) detected in oropharyngeal swabs (log_10_ copies/mL) and tissues (log_10_ copies/mg). **(f)** Individual viral titers in brain, lung, and nasal turbinate, expressed as log_10_ tissue culture infectious dose (TCID_50_)/mL; the dotted line indicates the assay’s limit of detection (10^1.8^ TCID_50_/mL). **(g)** SARS-CoV-2 nucleocapsid protein detection, and **(h)** histopathological lesion scores (0 = absent, 1 = low, 2 = moderate, and 3 = high) in the nasal turbinate, lung, and brain. Individual data points are shown in panels b-h along with the mean ± standard deviation (e-h).


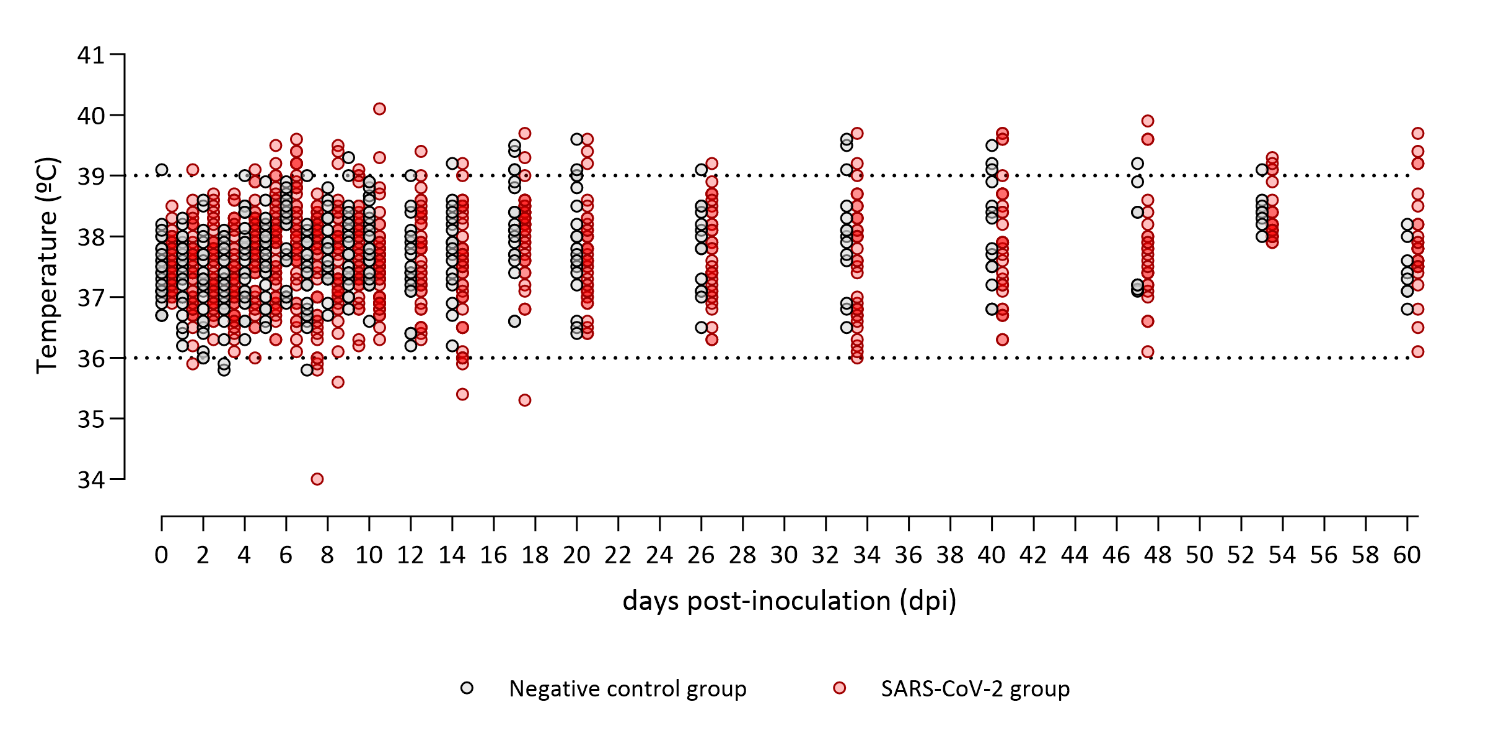


**Supplementary Figure S4. Subcutaneous temperature over time.** Individual temperatures (°C) for negative controls (gay squares) and SARS-CoV-2 inoculated mice (red rhomboids) are shown. Dotted lines indicate the normal physiological range (36-39 °C). No statistically significant differences were observed between groups.


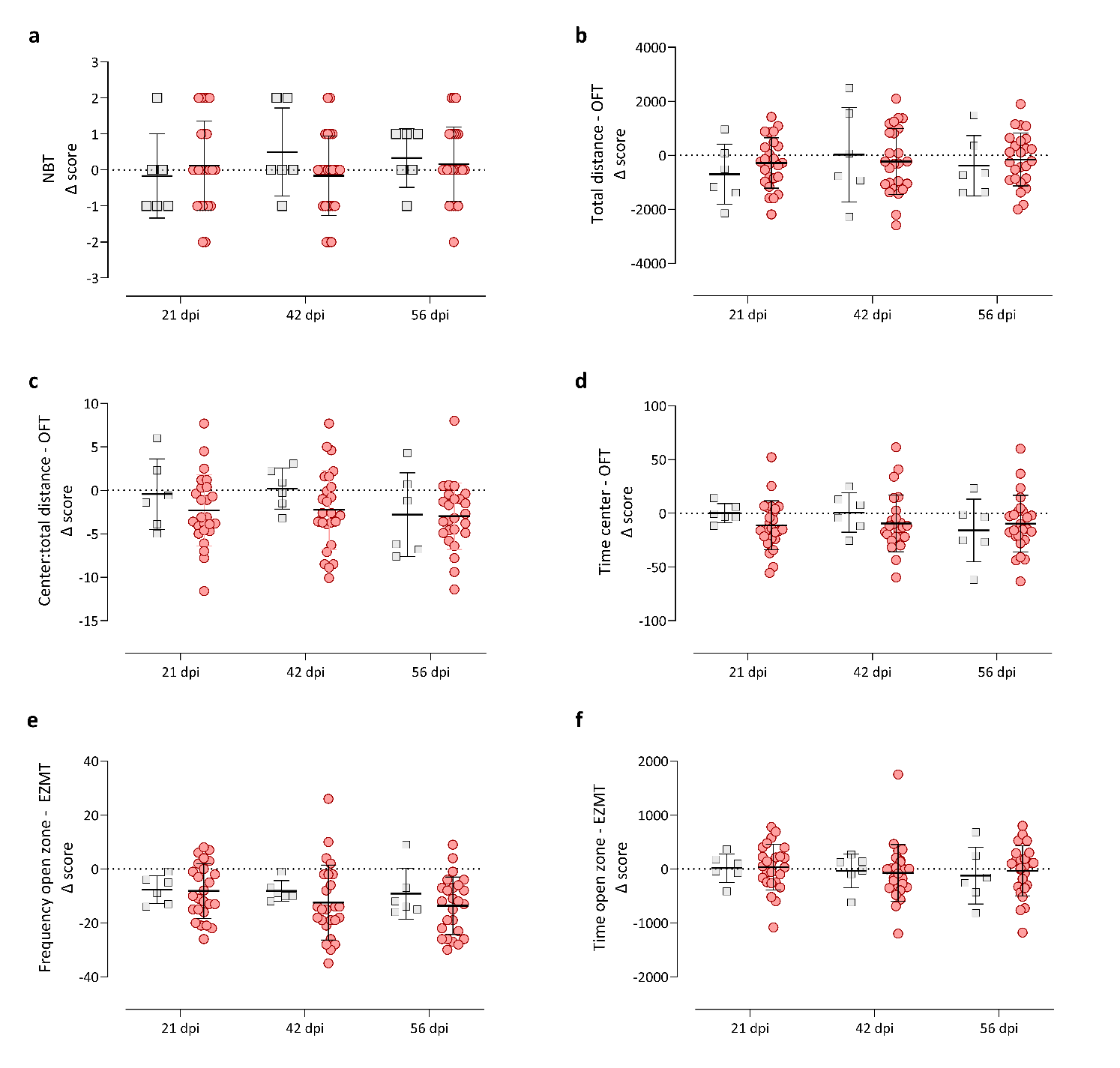


**Supplementary Figure S5. Behavioral delta (Δ) scores at 21, 42, and 56 days post-inoculation (dpi).** Behavioral performance was assessed using the **(a)** nest-building test (NBT); **(b)** total distance moved, **(c)** center-to-total distance ratio, and **(d)** time spent in the center during the open field test (OFT); as well as **(e)** frequency of entries into the open zone and **(f)** time spent in the open zone during the elevated zero maze test (EZMT). Δ scores were calculated relative to baseline values. Individual data points represent negative controls (gray squares) and SARS-CoV-2 inoculated mice (red circles), with mean ± standard deviation. No statistically significant differences were detected between groups or across time points.

**
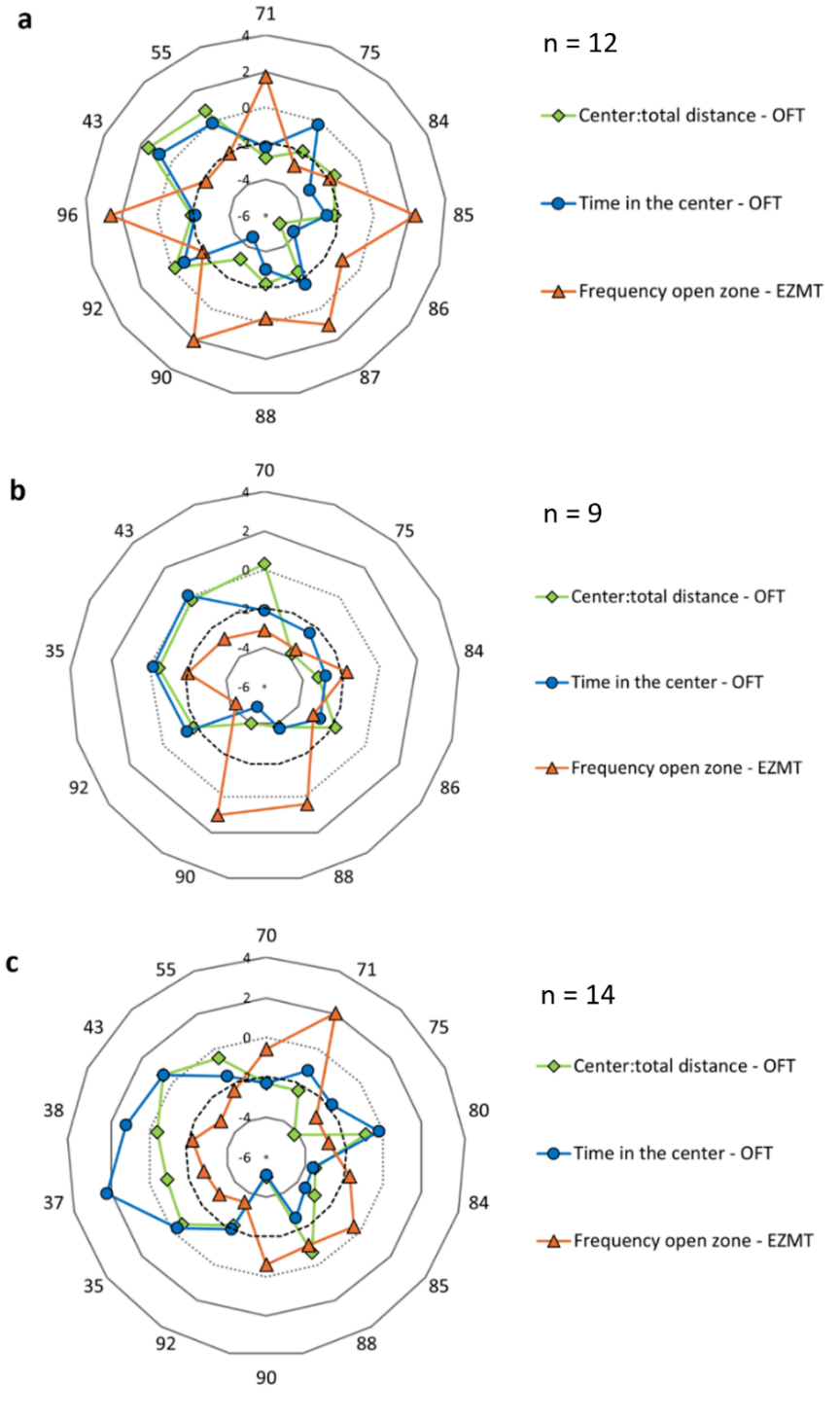
**

**Supplementary Figure S6. Behavioral performance at 21, 42, and 56 days post-inoculation (dpi).**

Z-scores derived from the open field test (OFT) and elevated zero maze test (EZMT) are shown at **(a)** 21 dpi, **(b)** 42 dpi, and **(c)** 56 dpi for male (ID 35-55) and female (ID 70-96) mice. Each vertex of the spider plot corresponds to an individual animal, identified by its ID number. Only animals exhibiting significant underperformance in at least one behavioral parameter are included in the plots. Represented behavioral parameters include center-to-total distance moved in the OFT (green rhomboids), time spent in the center of the OFT (blue circles), and frequency of entries into the open zone in the EZMT (orange triangles). Z-scores were calculated as the number of standard deviations by which each animal’s delta value (post-challenge minus pre-challenge) differed from the mean delta value of the control group. Values below 0 (dotted line) indicate performance lower than the control mean, and values below -1.96 (dashed black line) indicate statistically significant underperformance.


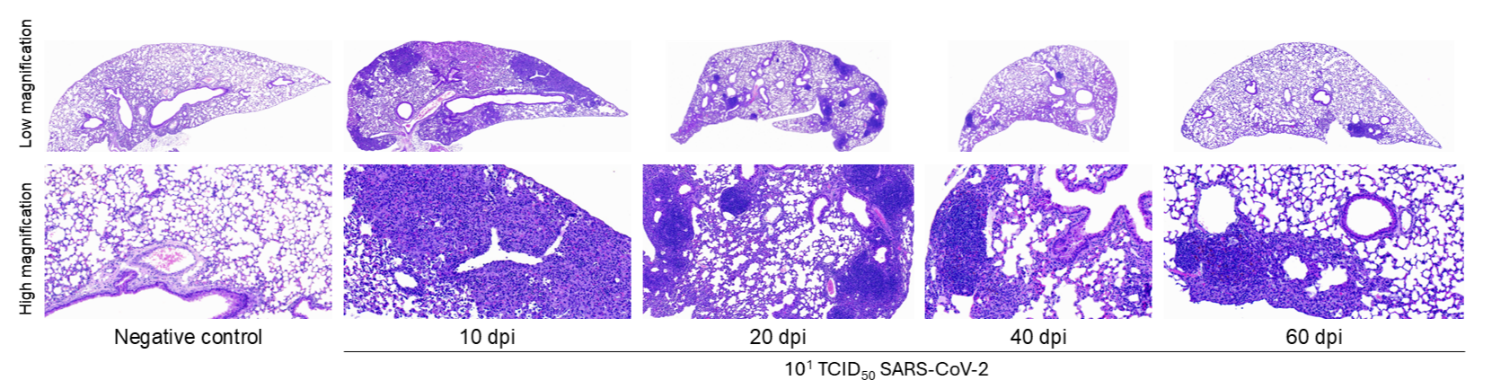
**Supplementary Figure S7. Representative lung histopathology over time.** Hematoxylin and eosin-stained lung sections from negative control and SARS-CoV-2 inoculated mice at 10, 20, 40, and 60 days post-inoculation (dpi), displayed at low magnification (top) and high magnification (bottom). In SARS-CoV-2 inoculated mice, multifocal broncho-interstitial pneumonia ranged from mild to moderate severity at 10 and 20 dpi and decreased to mild at 40 and 60 dpi, characterized by peribronchial lymphoid hyperplasia.


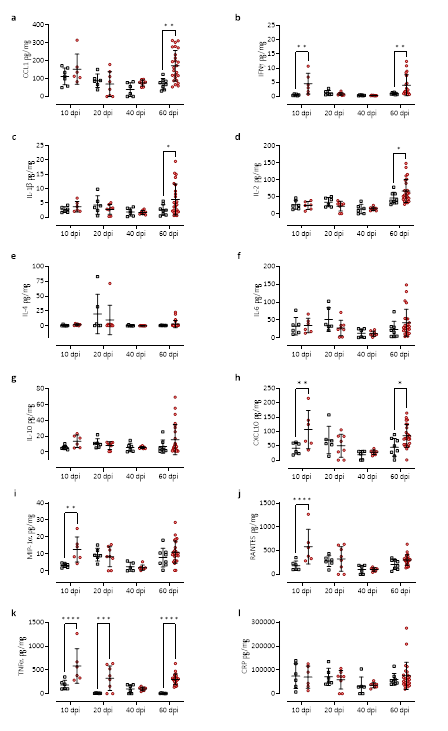


**Supplementary Figure S8. Lung cytokine profile following SARS-CoV-2 inoculation.** Lung cytokine concentrations (pg/mg protein) measured at 10, 20, 40, and 60 days post-inoculation (dpi): **(a)** CCL11, **(b)** IFNγ, **(c)** IL-1β, **(d)** IL-2, **(e)** IL-4, **(f)** IL-6, **(g)** IL-10, (h) CXCL10, **(i)** MIP-1α, **(j)** RANTES, **(k)** TNFα, and **(l)** CRP. Individual data points represent SARS-CoV-2-inoculated mice (n = 25, red circles), and negative control mice (n = 6, grey squares). Data are presented as mean ± standard deviation. Statistical analyses were performed using two-way repeated-measures ANOVA followed by Šídák’s multiple-comparisons test. Statistical significance is indicated as follows: p < 0.05 (*), p < 0.01 (**), p < 0.001 (***), and p < 0.0001 (****).


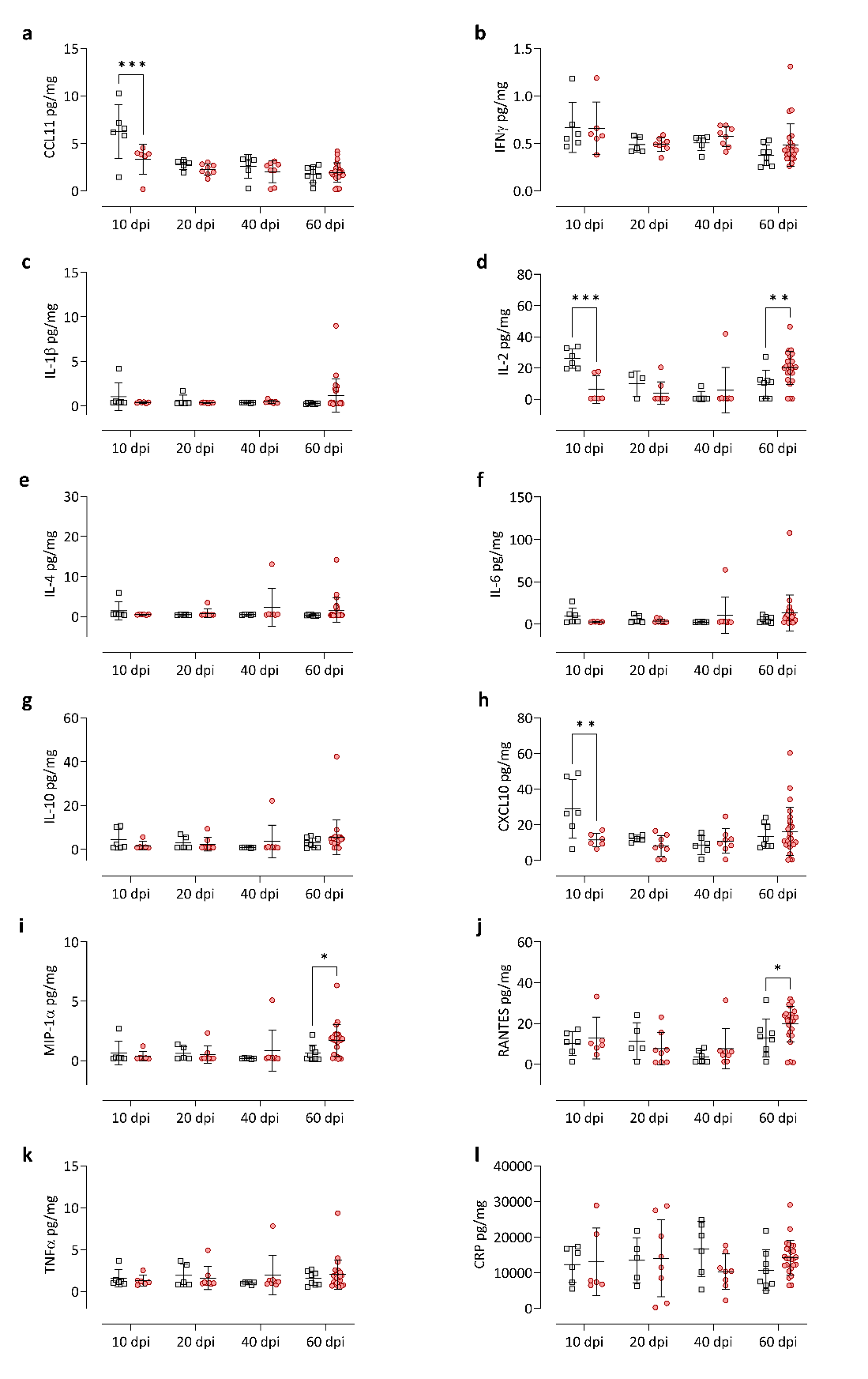


**Supplementary Figure S9. Longitudinal brain cytokine profile following SARS-CoV-2 inoculation.** Brain cytokine concentrations (pg/mg protein) measured at 10, 20, 40, and 60 days post-inoculation (dpi): **(a)** CCL11, **(b)** IFNγ, **(c)** IL-1β, **(d)** IL-2, **(e)** IL-4, **(f)** IL-6, **(g)** IL-10, (h) CXCL10, **(i)** MIP-1α, **(j)** RANTES, **(k)** TNFα, and **(l)** CRP. Individual data points represent SARS-CoV-2-inoculated mice (n = 25, red circles) and negative control mice (n = 6, grey squares), with means ± standard deviations. Statistical analyses were performed using two-way repeated-measures ANOVA with Šídák’s multiple-comparisons test. Statistical significance is indicated as follows: p < 0.05 (*), p < 0.01 (**), and p < 0.001 (***).

**
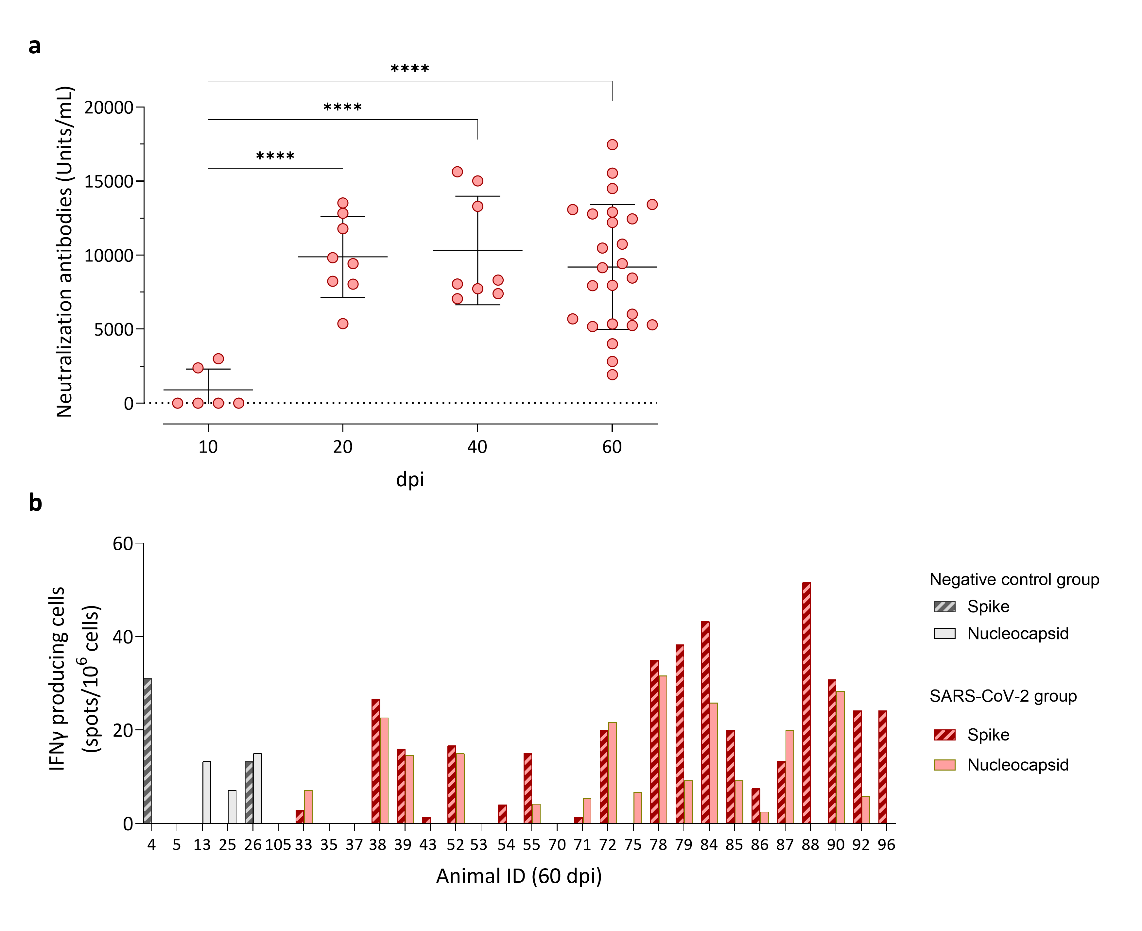
**

**Supplementary Figure S10. Humoral and cellular immune responses following SARS-CoV-2 inoculation. (a)** Neutralizing antibody levels (U/mL) in SARS-CoV-2 inoculated mice measured at 10, 20, 40, and 60 days post-inoculation (dpi). Statistical analyses were performed using two-way repeated-measures ANOVA followed by Šídák’s multiple-comparisons test. Statistical significance is indicated as follows: p < 0.001 (****). **(b)** Frequency of IFNγ-producing cells per 10^6^ splenocytes in negative controls (gray) and SARS-CoV-2 inoculated mice (red) at 60 dpi, following ex vivo stimulation with spike (dashed bars) or nucleocapsid (solid bars) proteins.

## Supplementary Tables

**Supplementary Table S1. Clinical scoring system for SARS-CoV-2 infection in K18-hACE2 mice.**

Clinical disease severity was assessed using a composite scoring system based on six parameters: neurological signs, locomotion, breathing, behavior, appearance, and body weight. Each parameter was scored on a scale from 0 (normal) to 3 (severe), with higher scores indicating greater severity. The total clinical score was calculated as the sum of all individual parameters. Animals reaching a cumulative score of ≥10 or a score of 3 in any single parameter met predefined humane endpoint criteria (HEP) and were euthanized.

| **Parameter** |  | **Score** |
| --- | --- | --- |
| **Neurological signs** | Normal | 0 |
|  | Mild tremors | 1 |
|  | Moderate tremors, ataxia | 2 |
|  | Seizures | 3 |
| **Locomotion** | Normal | 0 |
|  | Mild lameness/mild plantigradism | 1 |
|  | Moderate lameness or paresis/moderate plantigradism | 2 |
|  | Paralysis, immobility | 3 |
| **Breathing** | Normal | 0 |
|  | Increased or decreased respiratory frequency, nasal/ocular discharge | 1 |
|  | Moderate dyspnea | 2 |
|  | Severe dyspnea (gasping) | 3 |
| **Behavior** | Normal (alert) | 0 |
|  | Decreased activity or unusually docile when handled (apathy) | 1 |
|  | Stupor (unresponsive to environment and stimuli) | 2 |
|  | Coma (unresponsive to noxious stimuli) | 3 |
| **Appearance** | Normal | 0 |
|  | Mild piloerection/ungroomed, mild hunched posture | 1 |
|  | Moderate piloerection, moderate hunched posture, eyes partially closed | 2 |
|  | Severe piloerection, severe hunched posture, eyes closed | 3 |
| **Body weight** | ≥ 100% | 0 |
|  | 90-99% | 1 |
|  | 80-90% | 2 |
|  | < 80% | 3 |

**Supplementary Table S2. Correlations between peak acute clinical severity and post-acute outcomes.**

Pearson correlation coefficients (r) and corresponding p-values are reported for associations between the highest acute clinical score reached by each animal and post-acute parameters assessed at ≥ 21 days post-inoculation (dpi). Analyses are stratified by sex. Non-significant correlations are denoted as *n.s.* Statistical significance thresholds are defined as p < 0.05 (*), p < 0.01 (**), p < 0.001 (***).

| **Variable** | **dpi** | **Male r (p-value)** | **Female r (p-value)** |
| --- | --- | --- | --- |
| Neutralizing antibodies | 60 | 0.75 (0.0027; **) | 0.52 (0,0211; *) |
| Lung CCL11 | 60 | 0.56 (0.0406; *) | n.s. |
| Lung IL-6 | 60 | 0.78 (0.0014; **) | n.s. |
| Lung IFNγ | 60 | 0.72 (0.0046; **) | n.s. |
| Lung TNFα | 60 | 0.69 (0.0069; **) | n.s. |
| Brain CRP | 60 | n.s. | 0.50 (00321; *) |
| Vagus nerve CSA | 60 | -0.80 (0.0365; *) | n.s. |
| OFT: Center-to-total distance | 21 | n.s. | -0.60 (0.0063; **) |
| OFT: Center-to-total distance | 42 | n.s. | -0.53 (0.0207; *) |
| OFT: Time spent in center | 21 | n.s. | -0.82 (<0.0001; ***) |
| OFT: Time spent in center | 42 | n.s. | -0.53 (0.0194; *) |
| OFT: Time spent in center | 56 | n.s. | -0.46 (0.0450; *) |
